# Cell-type-specific requirement for TYK2 in murine immune cells under steady state and challenged conditions

**DOI:** 10.1007/s00018-025-05625-9

**Published:** 2025-03-02

**Authors:** Anzhelika Karjalainen, Agnieszka Witalisz-Siepracka, Michaela Prchal-Murphy, David Martin, Felix Sternberg, Milica Krunic, Marlies Dolezal, Nikolaus Fortelny, Matthias Farlik, Sabine Macho-Maschler, Caroline Lassnig, Katrin Meissl, Lena Amenitsch, Therese Lederer, Elena Pohl, Dagmar Gotthardt, Christoph Bock, Thomas Decker, Birgit Strobl, Mathias Müller

**Affiliations:** 1 Animal Breeding and Genetics, Department of Biological Sciences and Pathobiology, University of Veterinary Medicine Vienna, Vienna, Austria; 2 Present Address: Division Pharmacology, Karl Landsteiner University of Health Sciences, Krems an Der Donau, Austria; 3 Pharmacology and Toxicology, Department of Biological Sciences and Pathobiology, University of Veterinary Medicine Vienna, Vienna, Austria; 4 Physiology and Biophysics, Department of Biological Sciences and Pathobiology, University of Veterinary Medicine Vienna, Vienna, Austria; 5 Present Address: Department of Nutritional Sciences, Faculty of Life Sciences, University of Vienna, Vienna, Austria; 6 Campus Tulln, University of Applied Sciences Wiener Neustadt, Wiener Neustadt, Austria; 7 Platform Biostatistics and Bioinformatics, Department of Biological Sciences and Pathobiology, University of Veterinary Medicine Vienna, Vienna, Austria; 8 Department of Biosciences and Medical Biology, Center for Tumor Biology and Immunology, Paris-Lodron University Salzburg, Salzburg, Austria; 9 Department of Dermatology, Medical University of Vienna, Vienna, Austria; 10 Core Facility VetBiomodels, University of Veterinary Medicine, Vienna, Austria; 11 Cemm Research Center for Molecular Medicine of the Austrian Academy of Sciences, Vienna, Austria; 12 Institute of Artificial Intelligence, Center for Medical Data Science, Medical University of Vienna, Vienna, Austria; 13 Max Perutz Labs, Vienna Biocenter Campus (VBC), Vienna, Austria; 14 Center for Molecular Biology, Department of Microbiology, Immunobiology and Genetics, University of Vienna, Vienna, Austria

**Keywords:** JAK-STAT, Tonic signaling, Interferon, Splenic immune cells, Tumor-infiltrating cells, Macrophages, NK cells, CD8^+^ T cells

## Abstract

Tyrosine kinase 2 (TYK2) deficiency and loss or inhibition of kinase activity in men and mice leads to similar immune compromised phenotypes, predominantly through impairment of interferon (IFN) and interleukin 12 family responses. Here we relate the transcriptome changes to phenotypical changes observed in TYK2-deficient (*Tyk2*^−/−^) and TYK2 kinase-inactive (*Tyk2*^*K923E*^) mice in naïve splenic immune cells and upon ex vivo IFN treatment or in vivo tumor transplant infiltration. The TYK2 activities under homeostatic and both challenged conditions are highly cell-type-specific with respect to quantity and quality of transcriptionally dependent genes. The major impact of loss of TYK2 protein or kinase activity in splenic homeostatic macrophages, NK and CD8^+^ T cells and tumor-derived cytolytic cells is on IFN responses. While reportedly TYK2 deficiency leads to partial impairment of IFN-I responses, we identified cell-type-specific IFN-I-repressed gene sets completely dependent on TYK2 kinase activity. Reported kinase-inactive functions of TYK2 relate to signaling crosstalk, metabolic functions and cell differentiation or maturation. None of these phenotypes relates to respective enriched gene sets in the TYK2 kinase-inactive cell types. Nonetheless, the scaffolding functions of TYK2 are capable to change transcriptional activities at single gene levels and chromatin accessibility at promoter-distal regions upon cytokine treatment most prominently in CD8^+^ T cells. The cell-type-specific transcriptomic and epigenetic effects of TYK2 shed new light on the biology of this JAK family member and are relevant for current and future treatment of autoimmune and inflammatory diseases with TYK2 inhibitors.

**Supplementary Information:**

The online version contains supplementary material available at 10.1007/s00018-025-05625-9.

## Introduction

Tyrosine kinase 2 (TYK2) is a member of the Janus kinase (JAK) family of non-receptor protein tyrosine kinases and confers signal transduction induced by a variety of cytokines and growth factors, including type I interferons (IFN-I), interleukin (IL)−12, IL-22, IL-23 and, in a cell-type- or differentiation stage-specific manner, IL-10 and IL-13. Depending on stimulus, cell type and cellular context, TYK2-engaging receptor complexes are capable to activate all seven members of the signal transducer and activator of transcription (STAT) family [1]. Loss of TYK2 protein or its kinase activity in humans and mice causes high susceptibility to microbial infection, which is mainly caused by impaired production of IFNγ in response to IL-12 and IL-23 and defective IFN-I signaling [2–6]. TYK2 also exerts biological functions independently of its catalytic activity, which is best described for the cell surface stabilization of human IFNAR1 [4, 6]. In addition, kinase-independent functions have been reported in the context of signaling pathway cross-talks, basal mitochondrial functions [6, 7] and differentiation/maturation of cells [8, 9].

Cellular homeostasis refers to the processes involved in the maintenance of an intrinsic cellular steady state supporting viability and functionality [10]. Immune cells require constant alertness under homeostatic conditions for a rapid and adequate response to danger signals such as invading pathogens or tissue damage [11–14]. Mammalian immune cells do not rely on a simple switch between homeostatic maintenance and danger response. Rather, they use gradual signaling circuits with baseline activity under homeostatic conditions, rapid upregulation of key signaling components upon pathogen or damage recognition and return to steady state by counter-regulation [15–17]. IFN-I were shown to provide homeostatic alertness of the immune system [18, 19] and we reported TYK2 to be required for the maintenance of steady state levels of STAT1, a central constituent of the IFN responses [20].

Tumor surveillance or cancer immunity refers to the immune system's ability to specifically identify and eliminate tumor cells on the basis of their expression of tumor-specific antigens or molecules induced by cellular stress [21–23]. We have identified a crucial role of TYK2 in NK and T cell-mediated tumor cell elimination and in shaping the tumor microenvironment in various cancer models [24, 25]. A meta-analysis in human cancer patients confirmed the requirement for kinase-active TYK2 for a full-blown cancer immunity [26]. A kinase-independent function of TYK2 was found in NK cells, in which kinase-inactive TYK2 (TYK2^K923E^) restored in part NK cell maturation and cytotoxicity against tumor cells in vitro and in vivo [8].

In the present work, we analyzed the transcriptomes of *Tyk2*-deficient (*Tyk2*^−/−^) and *Tyk2* kinase-inactive (*Tyk2*^*K923E*^) splenic macrophages, NK and CD8^+^ T cells under steady state condition and upon IFNβ challenge [27]. In addition, we determined the cytolytic activity and the mitochondrial activity of *Tyk2*^−/−^ and *Tyk2*^*K923E*^ CD8^+^ T cells and NK cells and generated transcriptional profiles of both cell types upon tumor infiltration. We found that in splenic homeostatic and tumor-derived immune cells kinase-active TYK2 mainly impacts on the IFN response gene signature. Under both homeostatic and challenged conditions TYK2-dependent genes differed between cell types. In addition, we identified a novel role of TYK2 in transcriptional repression. Scaffolding functions of *Tyk2*^*K923E*^ CD8^+^ T cells and macrophages induced subtle changes of the transcriptome and the chromatin accessibility at promoter-distal regions upon IFNβ treatment, again in a cell-type-specific manner. Overall, we describe a novel and irreplaceable activity of TYK2 in IFN-induced gene repression, a gross cell type specificity of TYK2-dependent gene signatures and fractions of genes regulated independently of the kinase activity.

## Material and methods

### Mice

*Tyk2*^−/−^ (B6N.129P2-*Tyk2*^*tm1Bia*t^ or B6.129P2-*Tyk2*^*tm1Biat*^Tg(CMV-cre)1Cgn) [20, 28, 29] and *Tyk2*^*K293E*^ (B6.129P2-*Tyk2 *^*tm3.1(K923E)Biat*^) [30] were on C57BL/6N background and bred at University of Veterinary Medicine Vienna under specific pathogen-free (SPF) conditions according to FELASA guidelines. Housing conditions for the assessment of homeostatic RNA profiles are described in detail [27]. For the tumor transplant experiments *Tyk2*^−/−^ (B6N.129P2-*Tyk2*^*tm1Bia*t^) sex- and age-matched (6–12 weeks) mice were used. *Wild-type* (*WT,* C57BL/6N) mice were originally purchased from Janvier Labs.

### Purification, analysis and IFNβ treatment of primary splenic immune cells

Macrophages, NK cells and CD8^+^ T cells from three pooled spleens of *WT, Tyk2*^*K923E*^, and *Tyk2*^−/−^ mice were isolated, FACS enriched and either directly used for RNASeq or treated with IFNβ as described [27]. For CXCR3 staining spleen single cell suspensions were prepared by pressing through 100 μm cell strainers. NK cells were isolated using αDX5-labeled MACS® beads according to the manufacturer’s protocol (Miltenyi 130-052-501). Antibodies were purchased from eBioscience (NK1.1 [PK136], CD3ε [145-2C11]) and BD Biosciences (CXCR3 [CXCR3-173]). Analysis was performed with the BD FACSCanto II (BD Biosciences) and the BD FACSDiva software version 8.

### Metabolic extracellular flux assay

MACS beads-enriched splenic NK cells of *WT, Tyk2*^*K923E*^, and *Tyk2*^−/−^ mice were expanded in RPMI 1640 complete medium supplemented with 5000 U/ml IL-2 (Proleukin; Novartis) for 7 days. Purity of NK cells was assessed by flow cytometry and was typically 95–98% CD3ε^−^NK1.1^+^NKp46^+^ cells of all living cells on the day of the experiment.

Expanded NK cells were counted and resuspended in Agilent Seahorse XF RPMI medium (pH: 7.4, 5 mM Hepes; Table 1). The medium was supplemented with 1 mM pyruvate, 2 mM glutamine and 10 mM glucose for Mito Stress Test or only with 2 mM glutamine for Glycolysis Stress Test. Per well, 180 µl containing 250 k cells were loaded onto a Seahorse XFe/XF96 cell culture microplate pre-coated with Cell Tak according to manufacturer’s instructions (Corning, 14.8 µg/ml) followed by centrifugation at 200 *x*g for 2 min and subsequent resting for 45 min at 37 °C in a non-CO_2_ incubator. Oxygen consumption rates (OCR, pmol/min) and extracellular acidification rates (ECAR, mpH/min) were measured using the Seahorse XF-96 metabolic extracellular flux analyzer (Agilent). Oligomycin, carbonyl cyanide p-trifluoro-methoxyphenyl hydrazone (FCCP), rotenone and antimycin A (all from Sigma) were prepared as 2.5 mM stock solutions in DMSO (Sigma, cell culture grade) and diluted accordingly in the respective Seahorse XF RPMI Medium prior to the assay. After 15 min of equilibration, and measurement of basal respiration inside the XF-96 metabolic extracellular flux analyzer (37 °C), cells were subjected to 2 µM oligomycin (Injection 1), after 15 min to 1 µM FCCP (Injection 2) and finally to 1 µM rotenone and antimycin A (Injection 3) for additional 15 min. In the Glycolysis Stress Test, cells underwent exposure to 25 mM of glucose (first injection) for a duration of 15 min, succeeded by a 15-min treatment with 2 µM oligomycin (second injection), culminating in the administration of 50 mM 2-deoxy-glucose (third injection). The assays were stopped 30 min after the last injection. Oxygen related to the mitochondrial ATP production was calculated from the drop in OCR after blocking mitochondrial ATP synthase with oligomycin, in relation to basal respiration (OCR of the first 15 min of the assay). Glycolysis is given as the change ECAR following the addition of 25 mM glucose to cells deprived of glucose and pyruvate for one hour. The glycolytic reserve corresponds to the augmentation in ECAR subsequently to the inhibition of ATP synthase (using 2 µM oligomycin). Glycolytic capacity is determined by the ratio of the maximum achievable ECAR (induced by 2 µM oligomycin) to the basal non-glycolytic ECAR (observed after the introduction of 50 mM 2-deoxy glucose, which inhibits glycolysis).

**Table 1:** Reagents for agilent seahorse XF assays

Product	Stock	Manufacturer	Product number
Seahorse XFe96 FluxPaks		Agilent	102601-100
XF Seahorse medium		Agilent	103576-100
PBS		Sigma	D8537
Ethanol absolute, 99.9%		Carl Roth	9065.4
NaHCO_3_	0.1 M	Sigma-Aldrich	S6014-1 KG
Corning Cell-Tak Cell- and Tissue Adhesive pack	1.58 μg/μl	Corning	354,240
Pyruvate	100 mM	Agilent	103578-100
Glutamine	200 mM	Agilent	103579-100
Glucose	1 M	Agilent	103577-100

### T cell cytotoxicity assays

Preparation of SIINFEKL reactive T cells and the in vitro T cell cytotoxicity assay were performed as described previously [31]. In brief, peptide reactive or non-reactive T cells were co-cultured with carboxyfluorescein succinimidyl ester (CFSE, Molecular Probes, CellTrace™ CFSE Cell Proliferation Kit)-labeled OVA-expressing EG7 target cells in effector: target ratio 30:1, 15:1, 5:1 and 1:1. After 16 h the target cell lysis was assessed by flow cytometry.

The in vivo T cell cytotoxicity assay was performed as described previously [31]. In brief, mice were immunized by *s.c.* injection of m-TRP2181-188 (Bachem) and the adjuvant CpG-ODN 1668 (Eurofins). Seven days later they received *i.v.* syngeneic splenocytes which were pulsed with m-TRP2181-188, irrelevant peptide or left unpulsed, labeled with three different concentrations of CFSE and mixed in a 1:1:1 ratio. After 18 h draining lymph nodes were isolated and specific killing was assessed by flow cytometry.

### Purification of NK and CD8^+^ T cells from tumor transplants

Tumor transplants were performed with the MC38 adenocarcinoma cell line [32, 33] as described previously [29]. Briefly, 10^6^ MC38 cells were injected subcutaneously into each flank and tumor growth was monitored every other day. After 8–10 days tumors were isolated, weighed and cut tumors were digested in DMEM complete medium containing 1 mg/ml of collagenase D from Clostridium histolyticum (COLLD-RO, Sigma-Aldrich, cat. no. 11088858001) and 0.05 mg/ml of DNase I (Sigma-Aldrich, cat. no. 11284932001) for 1 h in 37 °C. The digested tissue was squeezed through 100 µm cell strainers twice to obtain single cell suspensions. CD45^+^ cells were enriched/isolated by magnetic separation using the MagniSortTM CD45 positive selection kit (Thermo Fisher Scientific Cat. No. 8802-6865).

### RNA-sequencing and ATAC-sequencing

RNA-seq and ATAC-seq experiments for naïve splenic immune cells and in vitro cultured short- or long-term IFNβ-treated cells were described previously [27]. For RNASeq of tumor transplants cells were stained for CD45 (30-F11), CD3ε (145-2C11), NK1.1 (PK136) and CD8a (53–6.7) and 300 NK cells (CD45^+^, CD3ε^−^, NK1.1^+^) or 300 CD8^+^ T cells (CD45^+^, CD3ε^+^, CD8a^+^) were sorted and lysed in buffer containing 0.2% Triton X-100 solution (Sigma-Aldrich, Cat. No. T9284) supplemented with RNAse inhibitor (1:20, Takara Clontech, 2313A). Samples were stored at −80 °C. RNA isolation, cDNA synthesis and RNASeq were performed as for homeostatic samples [27].

### RNA-seq analysis

Raw sequence quality check was performed using FastQC (Andrews S. (2010). FastQC: a quality control tool for high throughput sequence data. Available online at: http://www.bioinformatics.babraham.ac.uk/projects/fastqc). Fifty base pair long RNA single-end reads were trimmed with Trimmomatic (version 0.32) [34] and aligned to the *Mus Musculus* reference genome assembly version mm10 using STAR (version 2.7.1) [35]. Transcript quantification was performed by counting uniquely aligned reads in exons using the function summarizeOverlaps from the GenomicAlignments package (version 1.6.3) in R (version 3.2.3) [36]. Gene annotations were based on the Ensembl GENCODE Basic set (genome build GRCm38 release 93) [37]. Gene names were assigned using *org.Mm.eg.db* (Carlson M (2019). org.Mm.eg.db: Genome wide annotation for Mouse. R package version 3.8.2; DOI: 10.18129/B9.bioc.org.Mm.eg.db) The read counts were normalized by DESeq2 correcting for library size [38]. Differential expression analysis was performed using DESeq2. Genes with averaged normalized counts below 10 were excluded from further analysis. Genes were considered as significantly differentially expressed if the false discovery rate (FDR) was below a threshold of 0.05 and the absolute log2FC value was above or equal to 1. Count plots of single genes were generated using the DESeq2 function *plotCounts* that normalizes counts of a single gene by sequencing depth and adds a pseudocount of 1/2 to allow for log scale plotting. Heatmaps were generated using the R function *pheatmap* from pheatmap (version 1.0.12, https://cran.r-project.org/web/packages/pheatmap/index.html) on logarithm transformed count data (DESeq2 function *normTransform*), using euclidian distance for both rows and columns, and the complete clustering method. Plots were generated using ggplot2 (version 3.4.1, https://ggplot2.tidyverse.org).

### ATAC-seq analysis

Raw sequence quality check was performed using fastqc (Andrews S. (2010). FastQC: a quality control tool for high throughput sequence data. Available online at: http://www.bioinformatics.babraham.ac.uk/projects/fastqc). Fifty base pair long ATAC single-end reads were trimmed as for RNA-seq analysis and aligned to the *Mus Musculus* reference genome assembly version mm10 using bowtie2 (version 2.2.4) [39]. Primary alignments with mapping quality greater than 30 were retained. ATAC-seq peaks were called using MACS (version 2.7.6) [40] on each individual sample. Peaks were aggregated into a list of consensus peaks using the function *reduce* of the package GenomicRanges (version 1.38.0) in R (version 3.6.1) [36]. Consensus peaks that overlapped with blacklisted genomic regions (downloaded from https://github.com/Boyle-Lab/Blacklist/tree/master/lists) were discarded [41]. Quantitative measurements were obtained by counting reads within consensus peaks using the function *summarizeOverlaps* from the GenomicAlignments (version 1.22.1) package in R (version 3.6.1) [36]. Promoter regions were defined as the regions 200 bp upstream and 200 bp downstream of a transcription start site (TSS, ‘promoter’) and the enhancer regions were defined as the regions 3 kb and upstream and 3 kb downstream (‘promoter proximal enhancer’). Differential accessibility analysis was performed using DESeq2 as described for the RNA-seq data. Heatmaps were generated using the R function *heatmap2* from the package gplots (version 3.1.3). Plots were generated using ggplot2 (version 3.4.1).

### Gene set and functional terms enrichment analysis

Gene set enrichment analysis (GSEA) was performed using GSEA 4.1.0 against MSigDB v7.2 gene set collections: H Hallmark [42] on DESeq2 pre-ranked gene lists. Enriched gene sets were considered significant if their FDR was less or equal 0.05. Motif and functional terms enrichment analysis was conducted using homer v4.11 [43] on gene lists identified by DESeq2 and again used a FDR threshold of 0.05 to assess significance.

### Gene lists

The list of interferon-stimulated genes (ISG) and IFN-repressed genes (IRepG) was compiled based on the various resources including Interferome v2.0 [44], Reactome Knowledgebase [45], Gene Ontology (GO) [46], Molecular Signatures Database (MSigDB) [42] and the ISGs published by Mostafavi et al. [47]. IRepG were collected from [47–49]. Robust, tunable and common mammalian ISG [50, 51] were converted to murine orthologs using Ensembl (https://www.ensembl.org). The list of JAK-STAT signaling components was compiled according to the following reviews [24, 52, 53]. The list of NK and CD8^+^ T cell effector genes was compiled based on the following references [54–65]. Members of the gene families not mentioned in the reviews but expressed in mice were added to the gene list. The categorization of the gene families was done based on HUGO Gene Nomenclature Committee (HGNC, https://www.genenames.org) and checked for expression in mice with Ensembl (GRCm39), NCBI (Mus musculus), IGV (mouse GRCm38/mm10) [66].

### Validation of *Stat* expression by RT-qPCR

RNA from CD49b DX5^+^ MACS-purified NK cells (Miltenyi, 130-052-501) was isolated using peqGOLD TriFast reagent (PEQLAB, 30–2010), and reverse transcription was performed using iScript First Strand cDNA Synthesis Kit (Bio-Rad, 1708891BUN). The quantitative PCR was performed with a Stratagene MX3000 instrument (Agilent Technology, Boebelingen, Germany) using Ube2d2 as housekeeping gene as described [30].

The following primers were used: Ube2d2-forward 5′ -AGG TCC TGT TGG AGA TGA TAT GTT-3′, Ube2d2-reverse 5′-TTGGGAAATGAATTG TCA AGA AA-39, Ube2d2-probe 59-CCA AAT GAC AGC CCC TAT CAG GGT GG-3′.

The following QIAGEN assay kits were used: *Stat1* (Mm_Stat1_2_SG QuantiTect Primer Assa, QT01149519), *Stat2* (Mm_Stat2_1_SG QuantiTect Primer Assay, QT00160216), *Stat3* (Mm_Stat3_1_SG QuantiTect Primer Assay, QT00148750), *Stat4* (Mm_Stat4_1_SG QuantiTect Primer Assay, QT00103005).

### Additional statistical tests

One-way ANOVA with Bonferroni post hoc test was performed using GraphPad Prism® version 5.0, 6.0 or 8.0 for Mac (GraphPad Software). Statistical significances are indicated for each experiment (*p < 0.05, **p < 0.01, ***p < 0.001, ****p < 0.0001).

## Results

### TYK2 and kinase-inactive TYK2 impact on naïve immune cell transcriptomes in a cell-type-specific manner

TYK2 signaling has been extensively studied in various immune, inflammatory and cancer settings [1, 6, 24, 67]. A systematic and comparative analysis of the impact of TYK2 on the transcriptome of innate and adaptive primary immune cells has been only performed for peritoneal macrophages and splenic B cells [47, 68, 69]. A distinction between kinase-dependent and -independent effects of TYK2 on the transcriptome level has not been made.

**Fig. 1:**
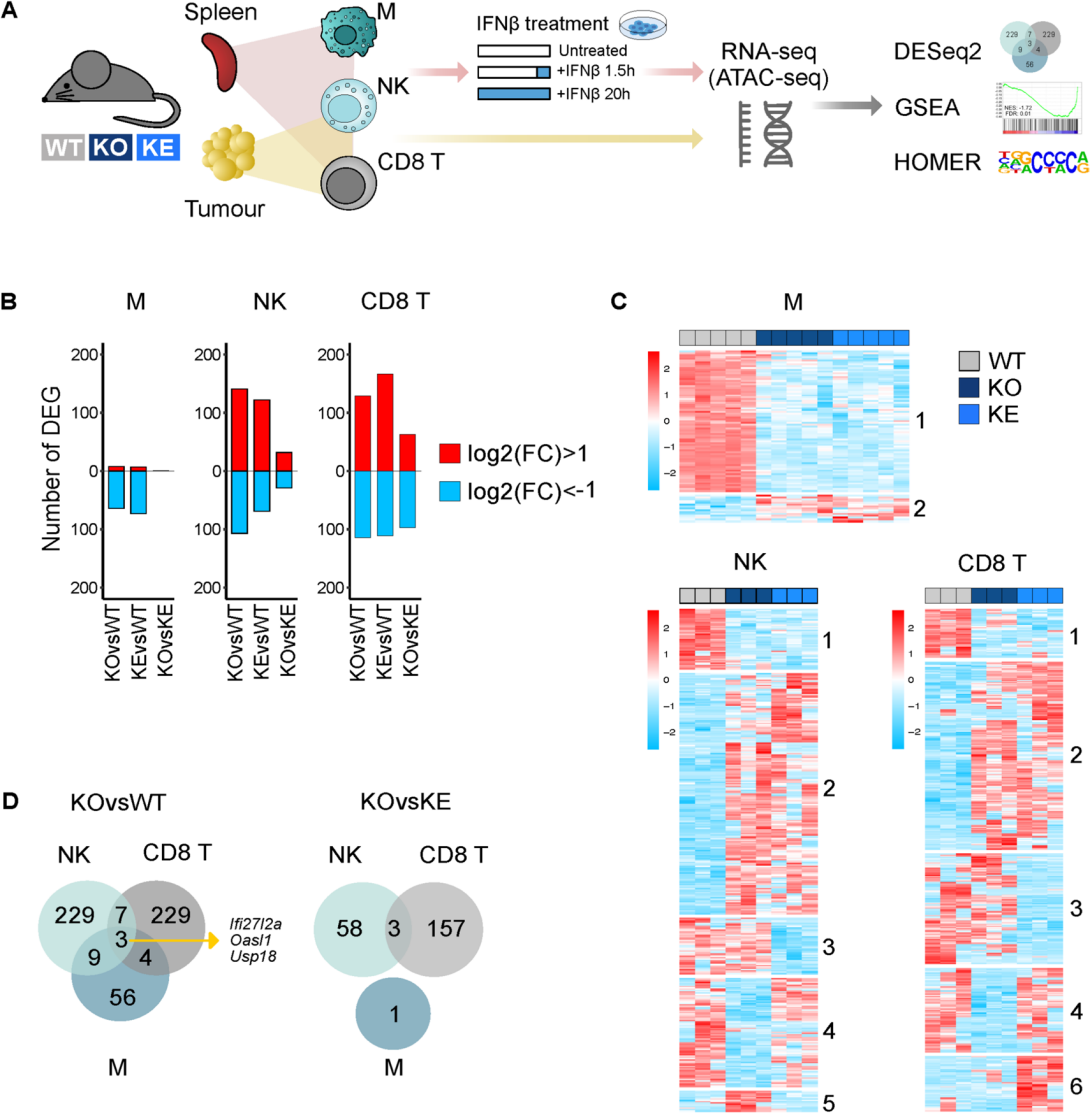

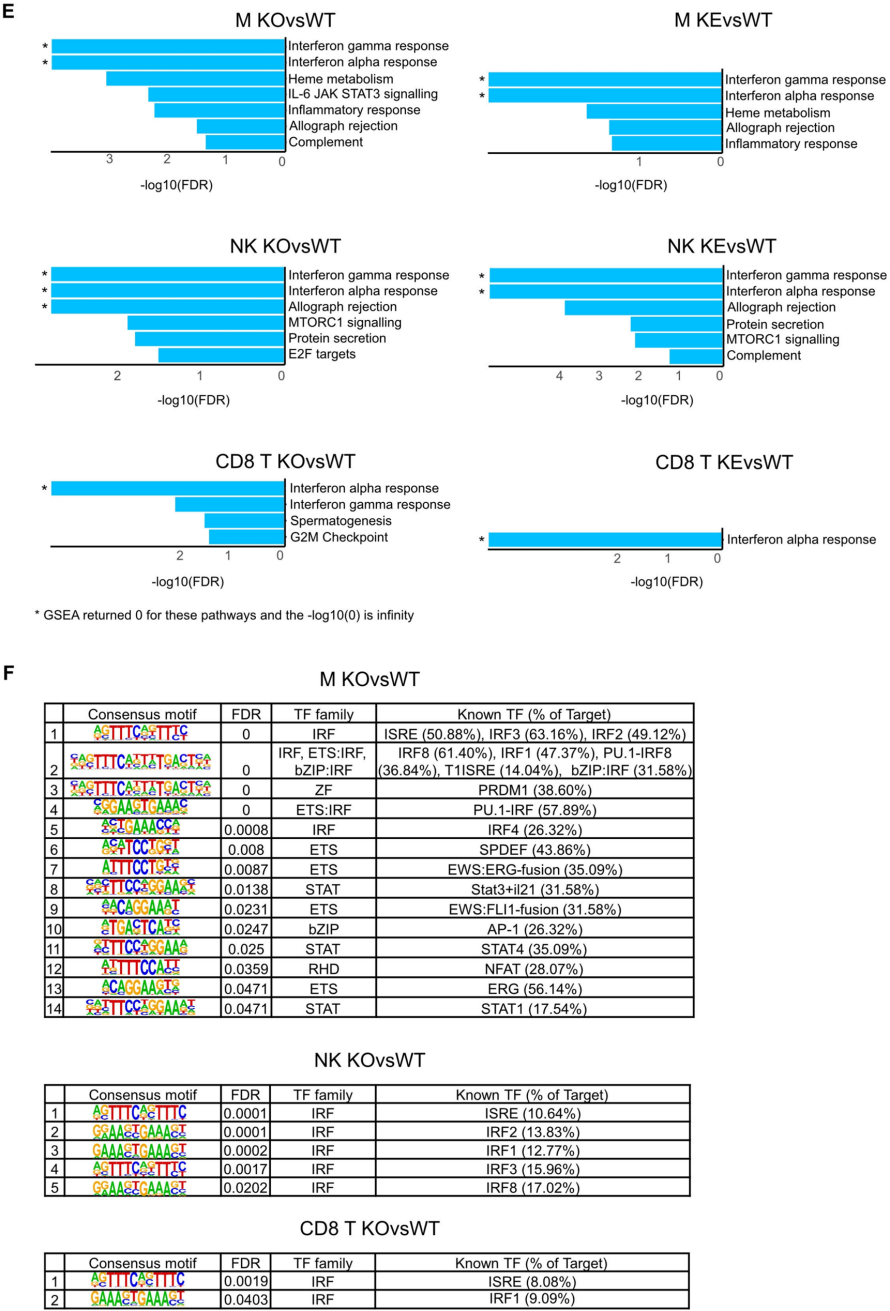
Experimental setup and transcriptional profiles of splenic macrophages, NK cells and CD8^+^ T cells upon loss of TYK2 or its kinase activity. **A** Scheme of experimental design with genetically modified mice (*WT*, wild type mice; KO, *Tyk2*^−/−^ mice; KE, *Tyk2*^*K923E*^ mice), collection of cells (M, macrophages; NK, natural killer cells; CD8 T, CD8^+^ T cells), treatments, next generation sequencing and computational analysis. **B** Number of significantly (|FC|> 2 or absolute log2(FC) > 1, FDR < 0.05) differentially expressed genes (DEG) determined by DESeq2 in the indicated genotype comparisons; upregulated (red) and downregulated (blue). **C** Heat map with scaled mRNA expression values (log2 cpm, counts per million) of DEG patterns (1–6). **D** Venn diagrams of genotype comparisons and cell type-specific DEG. **E** Highly represented pathways identified by gene set enrichment analysis (GSEA) against MSigDB v7.2 Hallmark pathways. False discovery rates (FDR) are indicated. *GSEA returned 0 and -log10(0) is infinity. **F** Hypergeometric Optimization of Motif EnRichment (HOMER) cis-regulatory motif discovery of TYK2-dependent DEG; FDR, transcription factor (TF) family and members and the percent of genes with the enriched motifs are indicated

This study builds on a systems-wide analysis of all STATs and other JAK signaling constituents in homeostatic and perturbed immune cells [27]. The impact of JAK-STAT on the transcriptomes was determined from splenic immune cells, since the largest secondary lymphoid organ governs a plethora of hematopoietic and immunological functions [70]. Bulk RNA-seq was performed of CD8^+^ T cells, NK1.1^+^ natural killer (NK) cells and F4/80^+^ macrophages FACS-purified from *WT*, *Tyk2*^−/−^ and *Tyk2*^*K923E*^ mice as representatives of the lymphoid and myeloid lineages. *WT* and *Tyk2*-mutant immune cell transcriptomes were also compared under IFN-treated and tumor-derived conditions (Figs. 1A, S1A [27],).

The effect of the *Tyk2* mutants on epigenetic cell states was assessed at the level of chromatin accessibility (ATAC-seq) for IFNβ-treated CD8^+^ T cells. In total, we in-depth analyzed transcriptome profiles of 104 samples and chromatin accessibility profiles of 26 samples (Table S1; [27]). To determine the effects of TYK2 and TYK2^K923E^ on transcriptomes and epigenomes of the cell types, differential expression (DE) and accessibility (DA) analyses were performed by comparing *Tyk2*^*−/−*^ vs *WT* cells (TYK2 knockout effect, KOvsWT), *Tyk2*^*K923E*^ vs *WT* (TYK2 kinase-inactive effect, KEvsWT) and *Tyk2*^*−/−*^ vs *Tyk2*^*K923E*^ (TYK2 mutations effect, KOvsKE) (Fig. 1B and following).

Based on the homeostatic spleen cell transcriptome datasets, we identified prominent cell-type- and mutant-specific gene expression patterns (Fig. 1B, Table S2). For both *Tyk2*-mutant mice macrophages showed a low number (> 90) of differentially expressed genes (DEG) which are nearly exclusively down-regulated (Fig. 1B, blue bars). The numbers of DEG in NK and CD8^+^ T cells were nearly threefold higher (> 200) and DEG showed up- and down-regulation. CD8^+^ T cells showed the highest effect of kinase-inactive TYK2 on DEG. Overall the impact of the TYK2 defects on up- or down-regulated DEG generates three distinct categories based on cell type and six patterns based on mutant-specific effects (Fig. 1C): (i) in all three cell types knockout and kinase-inactive effects overlap and show down-regulated (pattern 1) and up-regulated (pattern 2) DEG; (ii) in NK or CD8^+^ T cells knockout or kinase-inactive effects overlap with *WT* in up-regulated DEG (pattern 3 and 4, respectively); (iii) knockout or kinase-inactive effects only seen in NK cells (pattern 5) or in CD8^+^ T cells (pattern 6). Comparison of DEG in a cell-type- and genotype-specific manner revealed that the loss of TYK2 or its kinase activity affects only few genes common to all cell types. All shared protein-coding DEG are IFN-stimulated genes (ISG) (Fig. 1D**, **Table S2). The separation of DEGs in up- and down-regulated genes does not alter these findings (Fig. S1B).

To determine the effects of global TYK2 knockout and loss of kinase activity in the three cell types we performed Gene Set Enrichment Analysis (GSEA) [71] against MSigDB v7.2 Hallmarks gene set collection [42]. The top negatively enriched pathways (FDR ≤ 0.05) in all cell types and both genotypes were IFN alpha and gamma responses, except for the *Tyk2*^*K923E*^ CD8^+^ T cell transcriptome which only enriched for the IFN alpha response (Fig. 1E, Table S3), This confirms the central kinase-dependent role of TYK2 in the IFN pathways. Each cell type showed additional specific TYK2-dependent functions, notably with gross overlap upon loss of TYK2 protein or kinase activity (Fig. 1E, Table S3).

To further characterize the transcriptional signatures, we performed transcription factor (TF) motif enrichment in *cis*-regulatory elements of significantly DEG using Homer TF motif discovery (FDR ≤ 0.05; [43]) (Fig. 1F, Table S4). Consistent with the reduction in ISG expression in TYK2-mutant cell types, the most significant enrichments were for IFN-stimulated response elements (ISRE) and IFN regulatory factor (IRF) motifs (FDR = 0.0) which overlap [72]. While NK and CD8^+^ T cell signatures exclusively enriched for ISRE/IRF motifs, macrophages showed a broader spectrum of binding sites. These included motifs for TFs driving myeloid lineage differentiation and macrophage-specific functions (PRDM1/BLIMP-1 and ETS family or ETS:IRF) [73–75] as well as for STAT homodimers which is expected for an IFN signature but additionally supports dysregulated STAT3 signatures in the absence of TYK2 [76] (Fig. 1F, Tables S3, S4).

In summary, DEG analysis showed that the effect of loss of TYK2 or its kinase activity on the transcriptomes of NK and CD8^+^ T cells is substantially higher than in macrophages and that TYK2^K923E^ has the biggest effect in CD8^+^ T cells. In addition, TYK2 in macrophages acts mainly as an activator of transcription (down-regulated DEG) while in the lymphoid cell types TYK2 showed transcription maintaining and repressing (up-regulated DEG) functions. Common for all three cell types were the perturbed IFN pathways and DEG enrichment with ISRE/IRF/STAT(GAS) promoter binding sites in the absence of TYK2 kinase activity. TYK2 kinase-independent functions were most prominent in CD8^+^ T cells and detectable at the single gene level, while GSEA did not reveal gross switches in cell-type-specific pathways or known TF binding sites.

### TYK2 sustains a cell-type-specific pattern of tonically expressed ISG

To further evaluate the IFN signatures, we compiled an extensive list of more than 2000 genes (Table S5) that were previously reported as IFN-stimulated genes (ISG) from several resources (see Material and Methods). In addition, we included the emerging class of IFN-repressed genes (IRepG) [47–49] and tested our list against the transcriptomes of the three cell types.

**Fig. 2:**
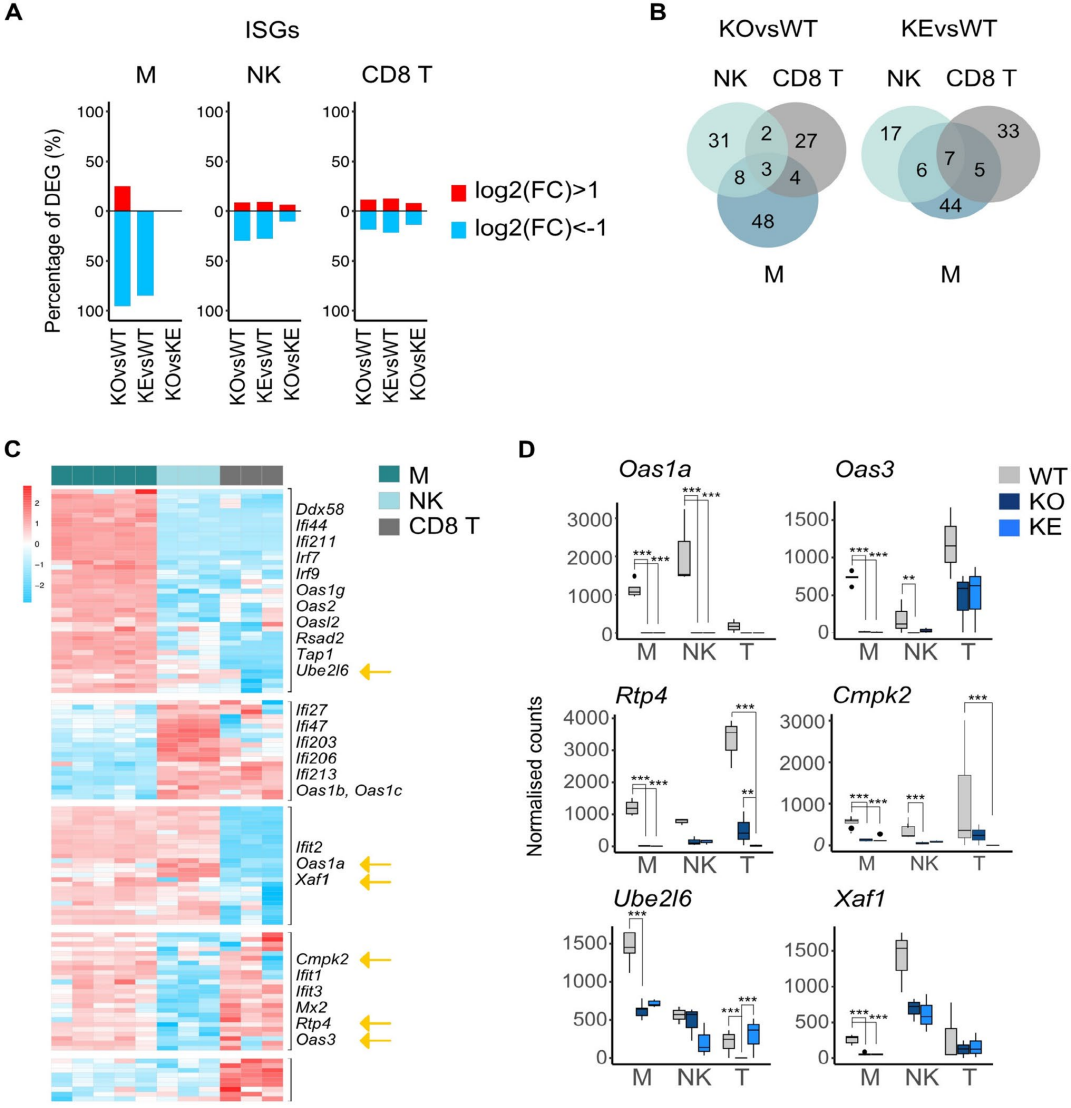
TYK2-dependent IFN signatures in splenic immune cells under steady state condition. **A** Percentage of IFN-stimulated genes (ISG), up- (red) or downregulated (blue) among DEG (|FC|> 2 or absolute log2(FC) > 1, FDR < 0.05). **B** Venn diagrams of genotype comparisons and cell type-specific DE ISG. **C** Heat map with scaled mRNA expression values (log2 cpm, counts per million) of DE ISG of *WT* macrophages, NK and CD8^+^ T cells, ‘robust’ ISG [50] named, arrows indicate ISG in (D). **D** Number of DESeq2 basemean reads aligned to ISG, mean and standard deviation are shown, *p < 0.05, ** p < 0.01, *** p < 0.001

Under steady state conditions TYK2 has pronounced, cell-type-specific effects on the ISG signature insofar as the DEG in macrophages are almost exclusively ISG while in NK and CD8^+^ T cells deregulated ISG are far less abundant (Fig. 2A). The TYK2-dependent ISG signatures had little communality between the cell types and a predominant kinase dependence (Fig. 2B).

Robust ISG are common to most mammalian cells, require only minute amounts of IFN, are activated even with low surface receptor levels and are driven by ISRE and IRF promoter elements [47, 50, 51]. At steady state, ISG (including robust ones) differed between *WT* cell types (Fig. 2C, robust ISG indicated). Genotype comparisons revealed a clear effect of TYK2 on maintenance of basal ISG expression in all cell types (Fig. S2A, pattern 1). In NK cells and CD8^+^ T cells kinase-inactive TYK2 restores tonic expression of few ISG (Fig. S2A, pattern 4). All three cell types also showed genes repressed by TYK2 (Fig. S2A, pattern 2). To test the possibility that the kinase-active TYK2 drives the robust gene signature, whereas minor changes in ISG expression are governed by TYK2^K923E^, we compared the respective lists [50, 51] of murine orthologs (Table S5) to the DEG of the three genotypes and cell types. No difference between the two classes of ISG with respect to the dependence on kinase-active TYK2 could be detected (Fig. S2B). Figure 2D shows selected ISG, which on top of the cell-type-specific expression in *WT* (grey box) show clear TYK2 effects (blue boxes) in one of the respective cell types.

In conclusion we report that in each cell type TYK2 impacts on tonic IFN responses and exerts its activity also in a cell-type-specific, i.e. context-dependent manner. Macrophages show the lowest number of DEG upon loss of TYK2 activity and nearly exclusively employ TYK2 for basal IFN responses. Tonic ISG signatures in *WT* cells show some overlaps between cell types but are also highly cell-type-specific even for genes previously described as robustly induced. This indicates that all ISG with detectable expression in one cell type require different intensity of signaling or varying TF activities for basal expression in another cell type.

### The JAK-STAT response machinery is differentially expressed in naïve *WT* cells and shows strongest dependency on TYK2 in NK cells

**Fig. 3:**
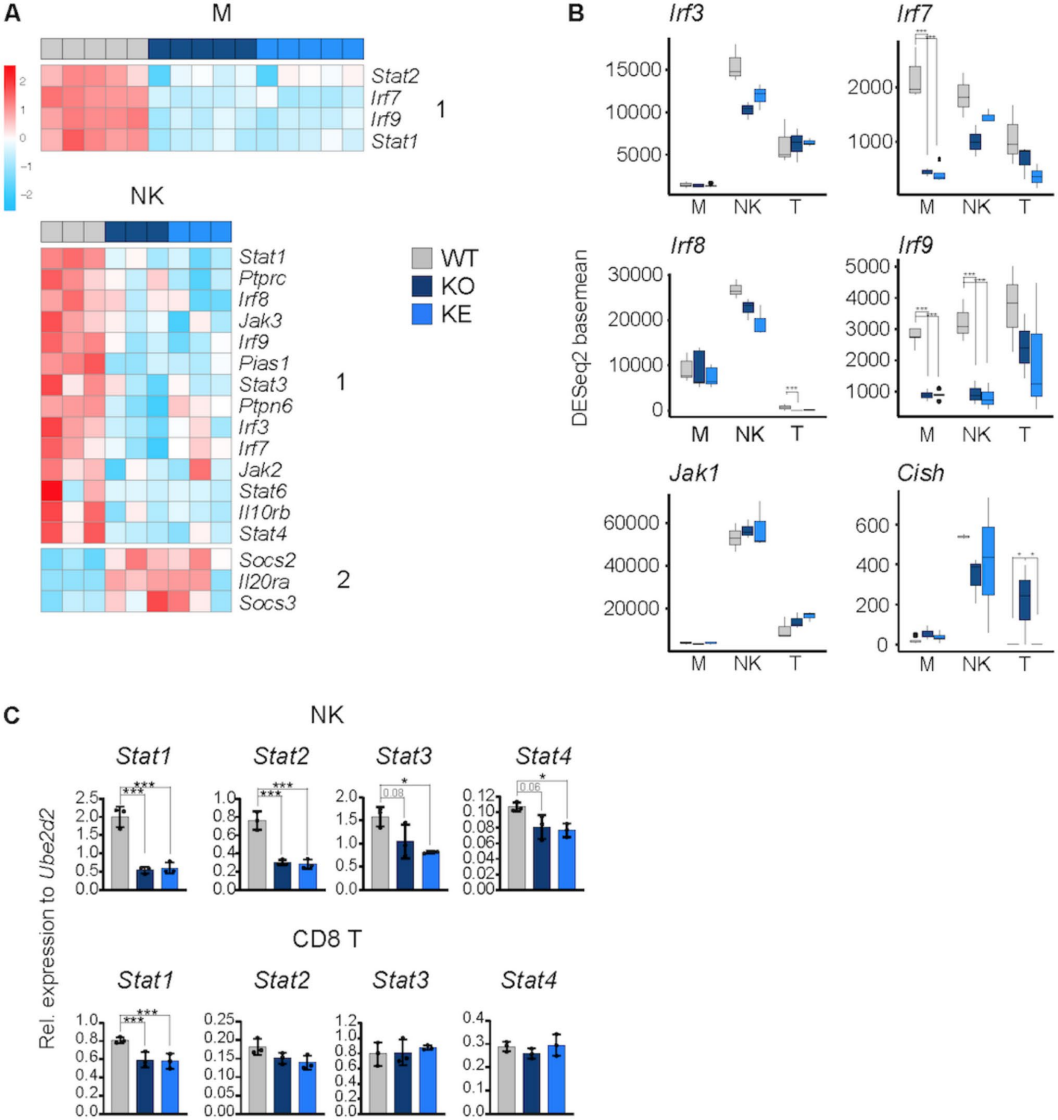
Expression levels of JAK-STAT signaling constituents under steady state conditions.** A** Scaled mRNA expression values of selected JAK-STAT signaling genes. **B** Number of DESeq2 basemean reads aligned to selected JAK-STAT signaling genes, mean and standard deviation are shown. **C** mRNA levels of selected of JAK-STAT signaling genes in NK and CD8^+^ T cells measured by RT-qPCR and shown as relative expression compared to the indicated housekeeping gene and given as mean ± SD. One-way ANOVA with Tukey multiple comparison, *p < 0.05, ** p < 0.01, *** p < 0.001

JAK-STAT responses are autoregulated by positive and negative transcriptional feedback loops, where STATs are capable to drive their own expression but also induce the expression of counter regulators, e.g. SOCS, PIAS or PTP proteins [77, 78]. Common JAK-STAT-SOCS signaling modules can have diverse differentiational and cell fate outcomes depending on the cytokine receptor activated and the spatial temporal accessibility of gene sets [79]. To analyze the JAK-STAT circuitry in WT cells and in dependence of TYK2 we generated a gene list of TYK2-engaging signaling components including signal promoting as well as suppressing components (see Materials and Methods and Table S6). All *WT* cells showed similar levels of *Tyk2* transcripts while *Jak1* transcripts were highest in NK cells, *Jak2* levels highest in macrophages and *Jak3* was least abundant in CD8^+^ T cells (Fig. S3 and Table S6). The relative *Stat1* levels were lowest in NK cells, *Stat2-6* transcripts were low in NK and CD8^+^ T cells, and *Stat4* expression was low in macrophages. Macrophages showed the highest relative expression of the STAT1-SOCS1 and the STAT3-SOCS3 modules, NK cells showed lowest expression of the major IFN-responsive TF complex ISGF3, which is composed of the subunits STAT1, STAT2 and IRF9, whereas CD8^+^ T cells appear to be equipped with low level of transcripts encoding counter regulators, including SOCS, PIAS and PTP family members (Fig. S3 and Table S6). As expected from the general transcriptional profile, TYK2 in macrophages drives the expression of the IFN response genes *Irf7,9* and *Stat1,2* (DEG pattern 1) and has otherwise little effect (Fig. 3A top panel). NK cells showed a considerable number of TYK2-dependent DEG (pattern1) at all levels of the response cascade, i.e. receptor chains, *Stats*, *Irfs* and counter regulators (*Ptpn6*, *Pias1*), which are reported ISGs (Table S5) or carry STAT binding sites in their proximal promoter regions [80]. Notably, the transcription of the STAT target genes *Socs2,3* and *Il20ra* seems to increase in the absence of kinase-active TYK2 (DEG pattern 2, IRepG), albeit only *Il20ra* reaches our statistical significance levels (Fig. 3A middle panel). CD8^+^ T cells do not depend on TYK2 for basal expression of the JAK-STAT constituents (Table S6). Figure 3B shows examples of cell-type- (*Jak1*, *Irf3,8*) and TYK2-specific (*Irf7,9*, *Cish*) effects on genes in the JAK-STAT signaling pathway. Impaired transcription of *Stat1-4* in TYK2-mutant NK cells and—except for *Stat1*—unperturbed expression of the selected STATs in CD8^+^ T cells was validated by RT-qPCR (Fig. 3C).

Taken together our comparison of JAK-STAT response components revealed cell type specificity of tonic expression levels and an impact of TYK2 on a variety of positive and negative feedback loop genes of the pathway, which provides one fundamental mechanism for the observed cell-type- and genotype-specific differences in gene expression. The predominant dependence of the tonic IFN response on TYK2 in macrophages is reflected by their exclusive loss of the feed-forward ISG *Stat1,2* and *Irf7,9*. While NK cells show a broad TYK2 dependence of the pathway circuitry, CD8^+^ T cells are grossly independent of TYK2 activity. The differential equipment with pathway components might be owed to their lineage specific requirements or reflect cell-type-, cellular localization- and/or genotype-specific differences under unperturbed conditions.

### The transcriptional profile of homeostatic *Tyk2*^−/−^ NK cells mirrors their functional impairment and is kinase-dependent

**Fig. 4:**
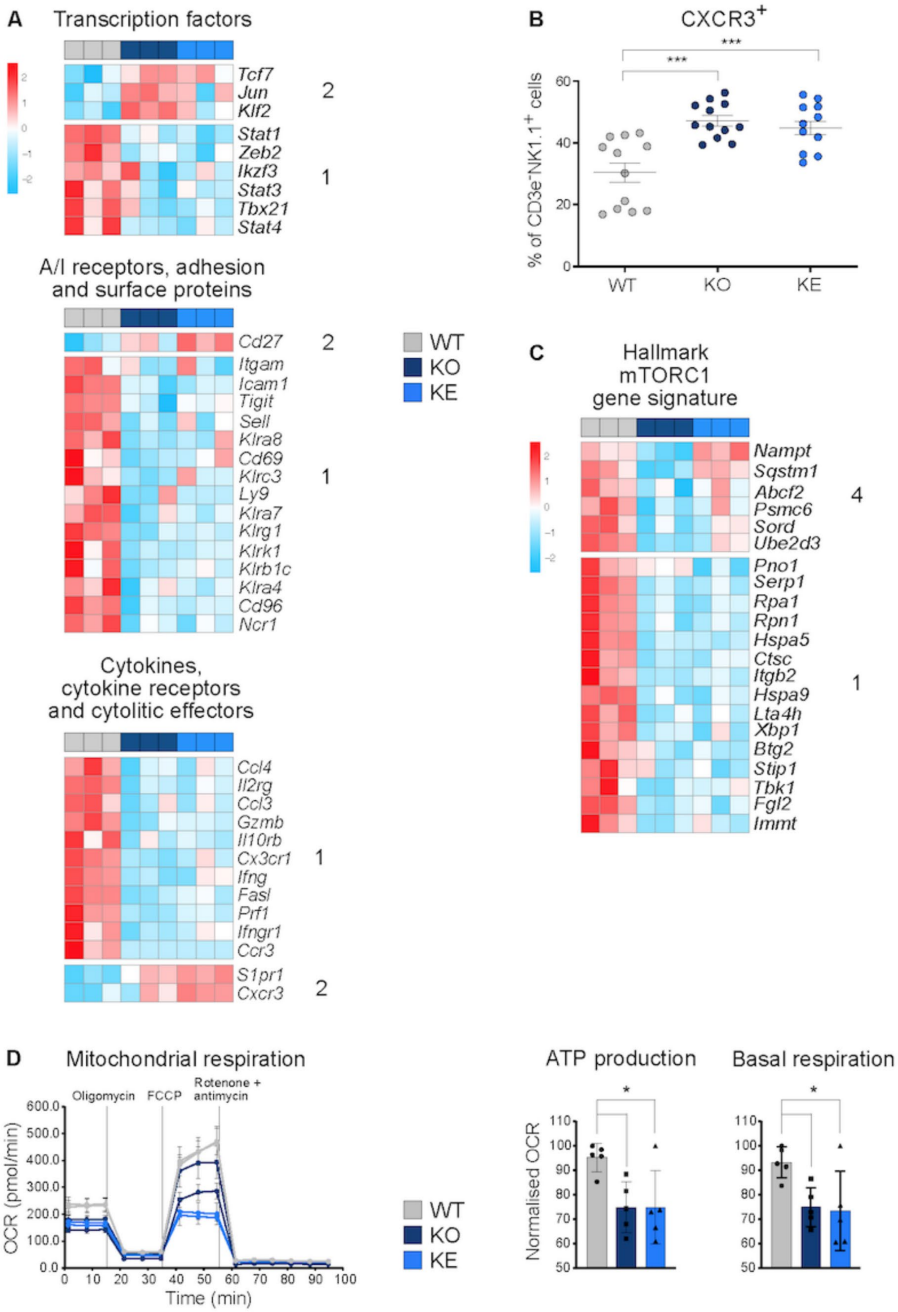
Transcriptional and functional profiles of splenic NK cells.** A** Scaled mRNA expression values of selected effector genes (log2 cpm, counts per million); A/I, activating/inhibitory; genotype patterns of DEG (1 and 2) are indicated. **B** CXCR3 cell surface expression of splenic NK cells of naïve mice analyzed by flow cytometry. Mean percentages ± SEM of CXCR3^+^ cells out of CD3ε-NK1.1^+^ cells from two independent experiments are shown; n = 12 for pooled experiments per genotype. *** p < 0.001. **C** Scaled mRNA expression values of leading genes in the mTORC1 signaling Hallmark. **D** Agilent Seahorse XF Cell Mito Stress Test of NK cells. NK cell representative oxygen consumption rate (OCR) curves are shown. Injections of oligomycin, FCCP, and rotenone + antimycin A are indicated by grey lines. NK cell ATP production and basal respiration were calculated for *WT*, *Tyk2*^−/−^ and *Tyk2*^*K923E*^ cells (n = 5) and are given as relative values compared to *WT* (100%). Two-way ANOVA with Tukey multiple comparison was used to assess statistical significance, *p ≤ 0.05

*Tyk2*-deficient mice show impaired NK cell maturation and cytotoxicity [81], which is grossly attributed to NK cell-extrinsic functions of TYK2 [82] and can—at least in part—be restored by kinase-inactive TYK2 [8]. So far, the analysis of TYK2^K923E^ in cytokine responses in various cell types has not revealed dominant negative or gain of function effects [30, 83, 84]. In line with these findings GSEA of DEG did not reveal indications for alternate JAK-STAT activities or a switch to other signal response pathways (Fig. 1E, Table S3). We analyzed the NK cell functions in detail by compiling lists of NK cell effector genes and testing them against the DEG of the mutants (Table S2, S6). The reduced maturation and cytotoxicity state of *Tyk2*-mutant NK cells [8] grossly correlates with the transcriptome data (Fig. 4A). Overall, most NK cell marker and effector RNAs in both *Tyk2*-mutant NK cells are down-regulated (pattern 1) while only few TF and cytokine-related genes are up-regulated in the mutants (Fig. 3A pattern 2). To further confirm the maturation defect of mutant NK cells we validated protein levels of CXCR3, which is preferentially expressed on immature NK cells [85]. Following the gene expression, *Tyk2*^*−/−*^ and *Tyk2*^*K923E*^ NK cells showed equally elevated levels of CXCR3 (Fig. 4B).

We previously reported an impact of TYK2 on cellular metabolism [86] and GSEA showed a significant negative normalized enrichment score (NES) for mTOR signaling in TYK2-mutant cells (Fig. 1E). A closer look at this hallmark gene signature revealed that some genes were expressed similarly to *WT* in a *Tyk2* kinase-independent manner (Fig. 4C pattern 4). Since mTOR regulates mitochondrial activities [87, 88] and kinase-inactive TYK2 suffices to maintain basic mitochondrial respiration in immune cells [89], we performed mitochondrial stress assays of in vitro expanded *Tyk2*-mutant NK cells. Figure 4D shows the mitochondrial dysfunction with respect to ATP production and basal respiration upon loss of TYK2 protein and kinase activity and preliminary data indicate a normal glycolytic performance (Fig. S4).

Summing up, the transcriptional profile of *Tyk2*^*−/−*^ NK cells explains to a substantial extend the cellular (dys-)functions while *Tyk2*^*K923E*^ transrciptome provides no explanations for functional restoration described previously [8, 30, 82]. TYK2 drives a basal mTOR signaling gene signature, however, in contrast to B cells [89], kinase-inactive TYK2 in NK cells does not restore loss of basal mitochondrial functions observed upon TYK2 deficiency. This indicates that also the kinase-independent functions of TYK2 are cell-type and/or context-dependent and TYK2—at least in NK cells—acts post-transcriptionally and/or -translationally.

### TYK2 kinase activity is required for CTL activity while the basal effector gene signature of naïve CD8^+^ T cells is TYK2-independent

Naïve CD8^+^ T cells from wildtype mice show varying expression levels of effector and marker genes, indicating that they receive varying extracellular cues and/or have differing threshold levels of intrinsic TFs to maintain basal transcription of these genes. In this cellular state, a vast majority of these genes does not depend on TYK2 or its kinase activity (Fig. S5A).

**Fig. 5:**
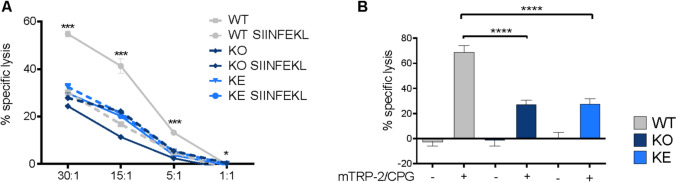
TYK2-dependent cytotoxicity of CD8^+^ T cells. **A** SIINFEKL peptide non-reactive (dashed line) or SIINFEKL peptide reactive (continuous line) T cells were co-cultured with CFSE-stained, OVA-expressing EG7 target cells at indicated effector:target ratios (30:1, 15:1, 5:1, 1:1) and after 16 h specific lysis was assessed by flow cytometry. Mean values ± SEM of technical replicates from one representative out of two independent experiments are shown. **B** *WT*, *Tyk2*^*−/−*^ and *Tyk2*^*K923E*^ mice were immunised with mTRP-2/CPG and after 7 days challenged with syngeneic splenocytes pulsed with mTRP2 peptide (CFSEhigh), pulsed with unspecific peptide (CFSEmid) or left unpulsed (CSFElow) at a 1:1:1 ratio. The specific killing was analysed in lymph node cell suspensions after 18 h by flow cytometry. Mean values ± SEM of technical replicates from one representative out of three independent experiments are shown; n = 5 per group. * p < 0.05, *** p < 0.001, **** p < 0.0001

We and others demonstrated that *Tyk2*-deficient CD8^+^ T cells lack cytotoxic activity [90, 91]. We thus tested whether kinase-inactive TYK2 is capable to confer this activity and found *Tyk2*^*K923E*^ CD8^+^ T cells to be indistinguishable from knockout cells with respect to their impaired in vitro and in vivo cytolytic capacity (Fig. 5A, B).

Notably, loss of *Tyk2* results in a negative NES of G2M cell cycle checkpoint genes which is not observed in *Tyk2*^*K923E*^ cells (Fig. 1E lower panel). Moreover, direct comparison of the mutant cells (KOvsKE) reveals an additional enrichment for E2F target genes governed by *Tyk2*^*K923E*^ (Table S4). Promoter analyses of DEG did not show other significant enrichments than the expected ISRE/IRF motifs (Fig. 1F). The cell cycle-specific signatures do not translate to an obvious shift in the numbers of detectable *WT* or mutant total T cells (CD3^+^) or CD8^+^ T cells in our FACS experiments (Fig. S5B, S5C). This is supported by our previous report of unperturbed CD8^+^ T cell proliferation in *Tyk2*-deficient mice upon activation by antigen presentation [91]. To further analyze the impact of TYK2 on the numbers of functional CD8^+^ T cell subsets [92, 93] we determined the share of effector and central memory T cells in the total CD8^+^ cells and again found no significant differences (Fig. S5B, S5C).

In conclusion TYK2 is not required for differentiation stage and cytotoxicity gene expression in CD8^+^ T cells. *Tyk2*^*K923E*^ CD8^+^ T cells show enrichment of cell cycle gene signatures compared to knockout which do not translate into an apparent altered activity state. In contrast to NK cells, *Tyk2*^*K923E*^ CD8^+^ T cells show no restoration of cytolytic activities.

### TYK2 is indispensable for an early and late IFNβ-repressed gene profile

**Fig. 6:**
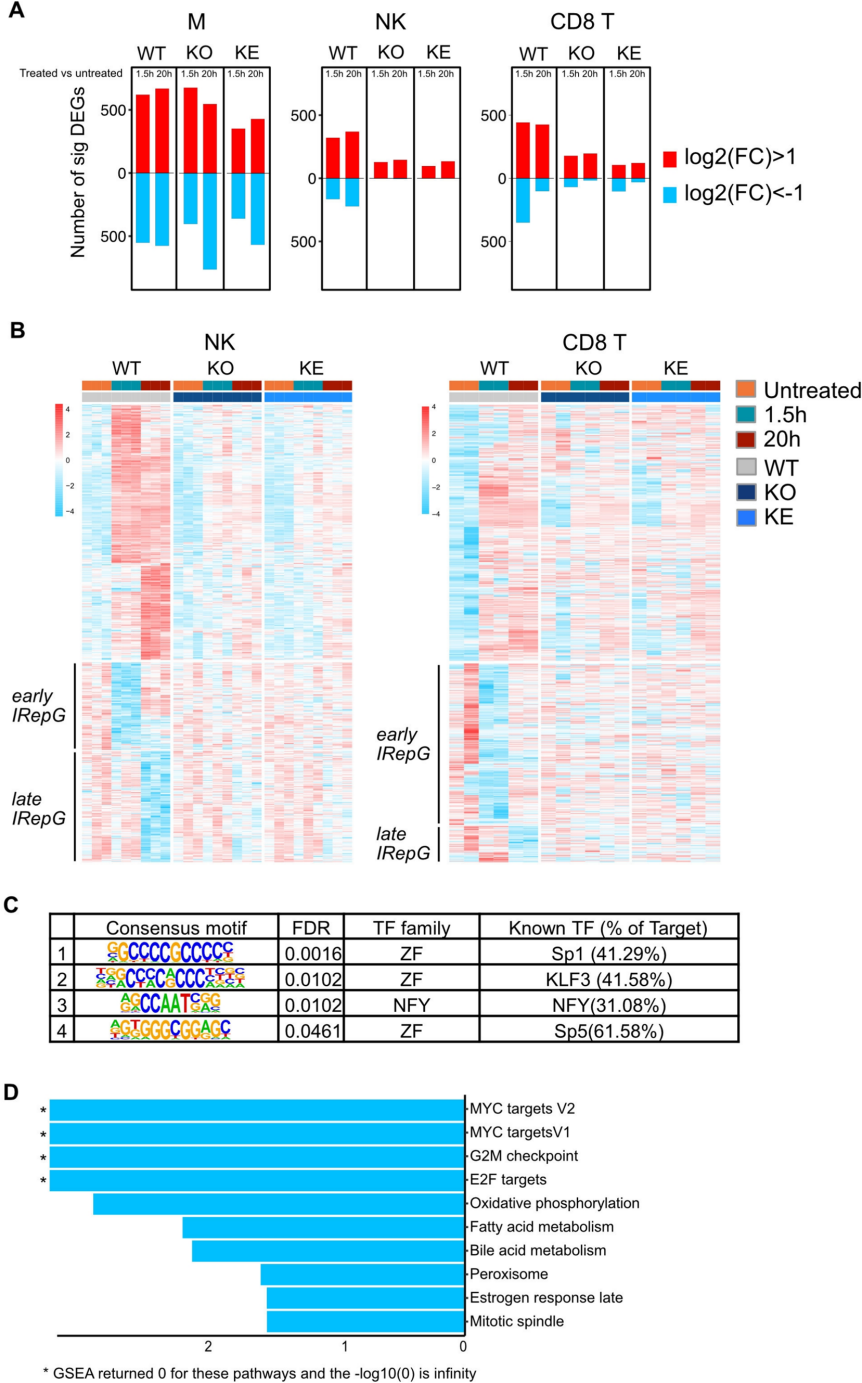
IFNβ treatments of in vitro cultured macrophages, NK and CD8^+^ T cells.** A** Number of significantly DE genes (|FC|> 2, FDR < 0.05) that are either upregulated (red) or downregulated (blue) at the indicated treatment time points as compared to untreated controls. **B** Scaled mRNA expression values of IFNβ-upregulated or repressed genes (log2 cpm, counts per million) in NK and CD8^+^ T cells after 1.5 h or 20 h treatment. **C** Hypergeometric Optimization of Motif EnRichment (HOMER) cis-regulatory motif discovery of IFNβ-repressed genes (1.5 h and 20 h IFNβ treatment combined) from NK and CD8^+^ T cells; FDR, transcription factor (TF) family and members and the percent of genes with the enriched motifs are indicated. **D** Gene set enrichment analysis (GSEA) of IFNβ-repressed genes in NK cells after 20 h treatment

IFN responses are tightly regulated by feed-forward and counter-regulatory mechanisms and hence show characteristic early and delayed gene activation patterns [72, 94, 95]. To cover both transcriptional activation states we treated in vitro cultured *WT* and *Tyk2*-mutant cell types short- and long-term with IFNβ (Fig. 1A). We previously reported that tissue context deprivation of immune cells leads to loss of gene signatures driven by JAK-STAT in situ and single IFNβ treatment restores multiple JAK-STAT pathways, including those not directly activated by the cytokine’s receptor [27]. Pairwise comparison of untreated versus treated *WT* and *Tyk2*-mutant cell types showed the biggest effect in macrophages (Fig. 6A, Table S2). All three *WT* cell types show at both IFNβ stimulation time points small overlapping gene sets (Fig. S6A), while the majority of response genes was cell-type-specific. The common ISG set greatly overlaps with the robust genes shown in Fig. 2C.

Here, we focused on the early and late IRepG, a signature feature seen in all *WT* splenic cells, albeit to a varying extend (Fig. 6A, Table S2). NK cells have one set of genes which is suppressed upon short-term treatment and re-expressed to the tonic level during long-term stimulus; a second set is suppressed only upon long-term treatment and does not change expression upon short-term stimulus (Fig. 6B). A similar pattern was observed in CD8^+^ T cells, albeit with a larger proportion of early suppressed genes which showed greater heterogeneity in the uninduced state and a smaller gene set which was repressed at long-term treatment (Fig. 6B). Macrophage IRepG at both treatment time points showed heterogeneity (Table S2). Notably, the transcriptional repressive function of TYK2 appears to be impartial, which contrasts its known partial effect on gene activation [20, 96]. In accordance with our previous observation on up-regulated ISGs [30], kinase-inactive TYK2 had no effect on the IRepG (Figs. 6B, S6B). The IRepG signature shows some ‘common’ genes but is more cell-type-specific than the IFN-induced signature (Fig. S6A). The comparison of our data set with the published IRepG (Table S5) shows a substantially expanded gene list especially for the long-term IFN treatment (Table S2).

To gain insight in the regulation of the IRepG we performed proximal promoter analysis of all down-regulated DEG of NK cell and CD8^+^ T cell IRepG. In accordance with previous predictions [49], we found a strong enrichment for SP1 TF binding and additional for KLF3, NFY and SP5 motifs (Fig. 6C**, **Table S4). GSEA of DEG revealed a significant negative NES for cell cycle and metabolic processes in NK cells at the late time point of IFNβ treatment (Fig. 6D, Table S3). Extending the functional enrichment analysis to HOMER [43] by retaining the GO terms with a gene count threshold of 2 as recommended [97] revealed IRepG enrichment for pathways ‘gene expression’, ‘post-translational modification’, ‘signaling through G protein-coupled receptors (GPCR)’ and ‘cytokine/interleukin signaling’ in NK and CD8^+^ T cells after brief IFNβ stimulation and in long-term IFN-treated NK cells. Owed to the small size of IRepG, CD8^+^ T cells showed no GO terms (Table S4).

Of note, the initial identification of IRepG was in the IFNγ response and not in splenic immune cells [48, 49]. However, we and others have shown that type I and II IFN have a large overlap in their potency to induce or repress ISGs in various cell types [47, 98–101].

Summing up, our data confirm the partial TYK2 dependence of most IFN-inducible genes, which, in analogy to the steady state condition, is cell-type-specific. This is the first notion of the absolute requirement of TYK2 kinase activity for a cell-type-specific early and late IRepG signature. The early IRepG are over-represented in the categories ‘suppression of cytokine/chemokine/growth factor signaling’ and ‘general gene expression’ and likely function to interfere with microbial replication or spread [49, 102]. The late IRepG are over-represented in the category ‘inhibition of cell cycle and metabolism’ and go in line with the well described antiproliferative, apoptotic and metabolic activities of ISG [103–106].

### Tumor-infiltrating NK and CD8^+^ T cells depend on kinase-active TYK2 for the expression of gene signatures induced by the tumor microenvironment

**Fig. 7:**
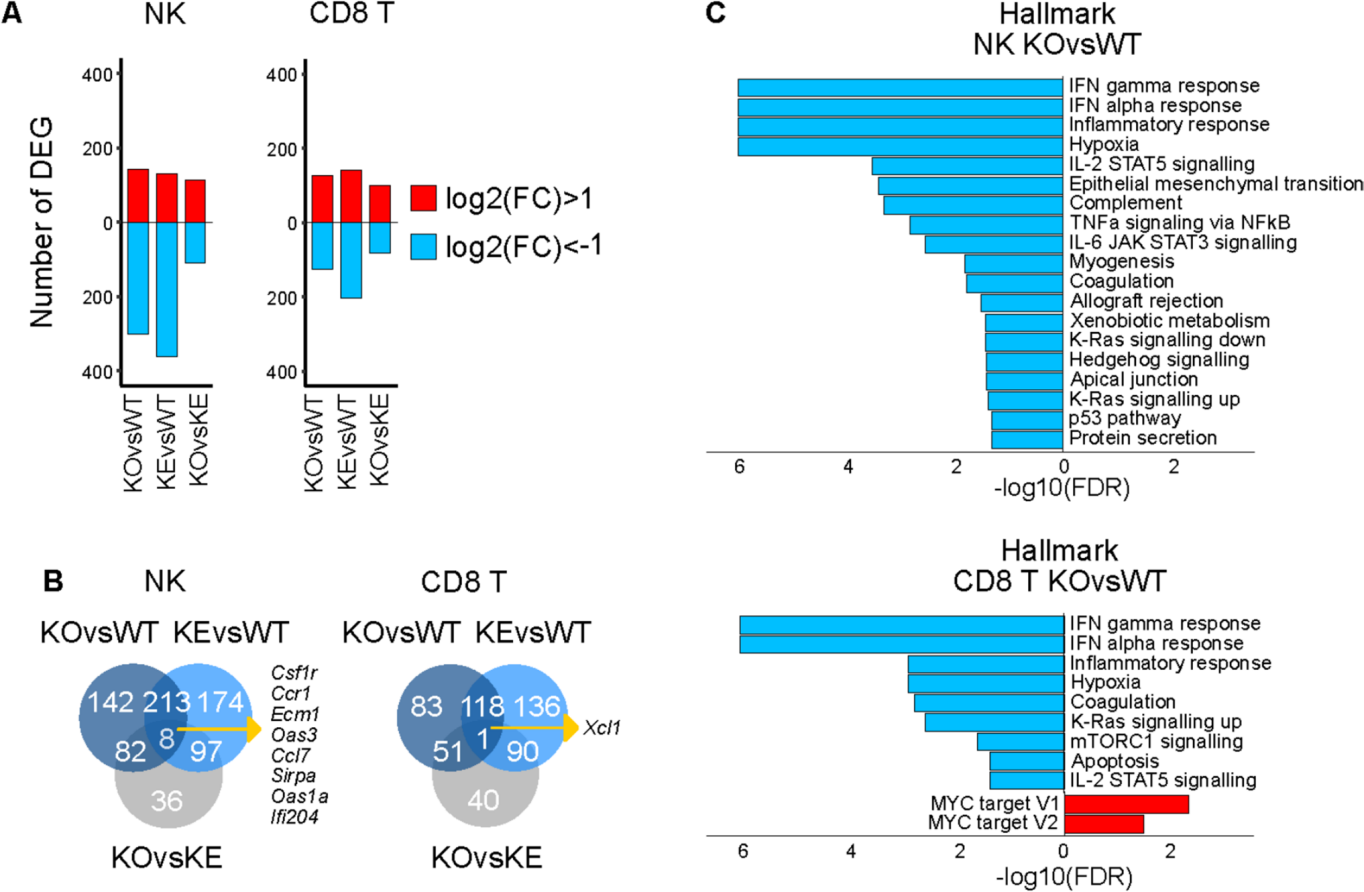
Transcriptomes of tumor-infiltrating *Tyk2*-deficient NK and CD8^+^ T cells.** A** Number of significantly (|FC|> 2 or absolute log2(FC) > 1, FDR < 0.05) differentially expressed (DE) genes determined by DESeq2 in the indicated genotype comparisons; upregulated (red) and downregulated (blue). **B** Venn diagrams of genotype comparisons of DEG in NK and CD8^+^ T cells. **C** Gene set enrichment analysis (GSEA) of NK or CD8^+^ T cells DEG from indicated comparisons; positive normalized enrichment scores (NES) (red) and negative NES (blue)

We previously established the requirement for TYK2 in various tumor surveillance settings [29, 81, 82, 91, 107]. Therefore, we determined the transcriptomes of *Tyk2*-deficient tumor-derived NK and CD8^+^ T cells and asked whether the observed functional restoration of *Tyk2*^*K923E*^ NK cells [8] becomes apparent at the transcriptional level upon exposure to the complexity of the tumor microenvironment. We used the MC38 adenocarcinoma transplantable tumor model previously established in *Tyk2*^−/−^ mice [29] since it allows simple separation of tumor-infiltrating CD45^+^ hematopoietic cells from tumor cells. The total number of significantly downregulated DEG in *Tyk2*^−/−^ and *Tyk2*^*K923E*^ NK cells was approximately twofold higher than in tumor-infiltrating CD8^+^ T cells (Fig. 7A). The *Tyk2*-mutant effect in NK and CD8^+^ T cells showed a substantial overlap with 213 and 118 genes, respectively (Fig. 7B), indicating no gross impact of kinase-inactive TYK2 on the transcriptome of both cell types. Notably, the genes common for all genotype comparisons in both cell types are all ISG, i.e. direct targets of the TYK2-STAT1/3 axis (Table S5) [24]. This is in line with GSEA providing for both mutant genotypes and cell types ‘IFN gamma’ and IFN alpha response’ as top negative NES (Fig. 7C, Table S4).

All mutant cells showed loss of characteristics of *WT* tumor-infiltrating cytolytic immune cells (Fig. 7C), i.e. hypoxia response [108], metabolic activities [109] and various signaling modules [110, 111]. The detailed analysis of NK cell marker and effector genes as well as JAK-STAT signaling components revealed no gross changes in the transcriptional pattern compared to the homeostatic or IFN-induced conditions (Fig. S7A, S7B left panels). The transcriptome of tumor-derived *Tyk2*^*K923E*^ NK cells does not reflect their in vitro and in vivo anti-tumor activity [8]. As expected from the results of the cytotoxicity assays TYK2-mutant tumor-derived CD8^+^ T cells also show no striking differences in the expression of effector or JAK-STAT genes (Fig. S7A, S7B right panels). Notably, *Tyk2*-mutant CD8^+^ T cells and *Tyk2*^*K923E*^ NK cells show a positive NES for hallmark MYC targets (Figs. 7C, S7C). *Tyk2*^*K923E*^ CD8^+^ T cells show negative NES of hallmarks G2M checkpoint and E2F targets (Table S3). This goes along with the notion that *WT* cells suppress these signatures upon IFN treatment (see above). As expected from the prominent IFN signatures of tumor-infiltrating NK and CD8^+^ T cells, promoter analysis of significantly DEG revealed enrichments for ISRE and IRF motifs in both cell types (Fig. S7D).

In conclusion, the exposure of *Tyk2*-deficient NK and CD8^+^ T cells to the multiple soluble and cellular stimuli during tumor infiltration shows the transcriptional profiles expected for the previously described functional impairment of the cells. As observed for the other conditions, TYK2^K923E^ in both cell types does not induce transcriptional profiles indicative for the restoration of *WT* or for the gain of novel functions.

### Chromatin accessibility and transcriptional profile of IFNβ-treateed CD8^+^T cells overlap and TYK2^K923E^ allows for chromatin remodeling

**Fig. 8:**
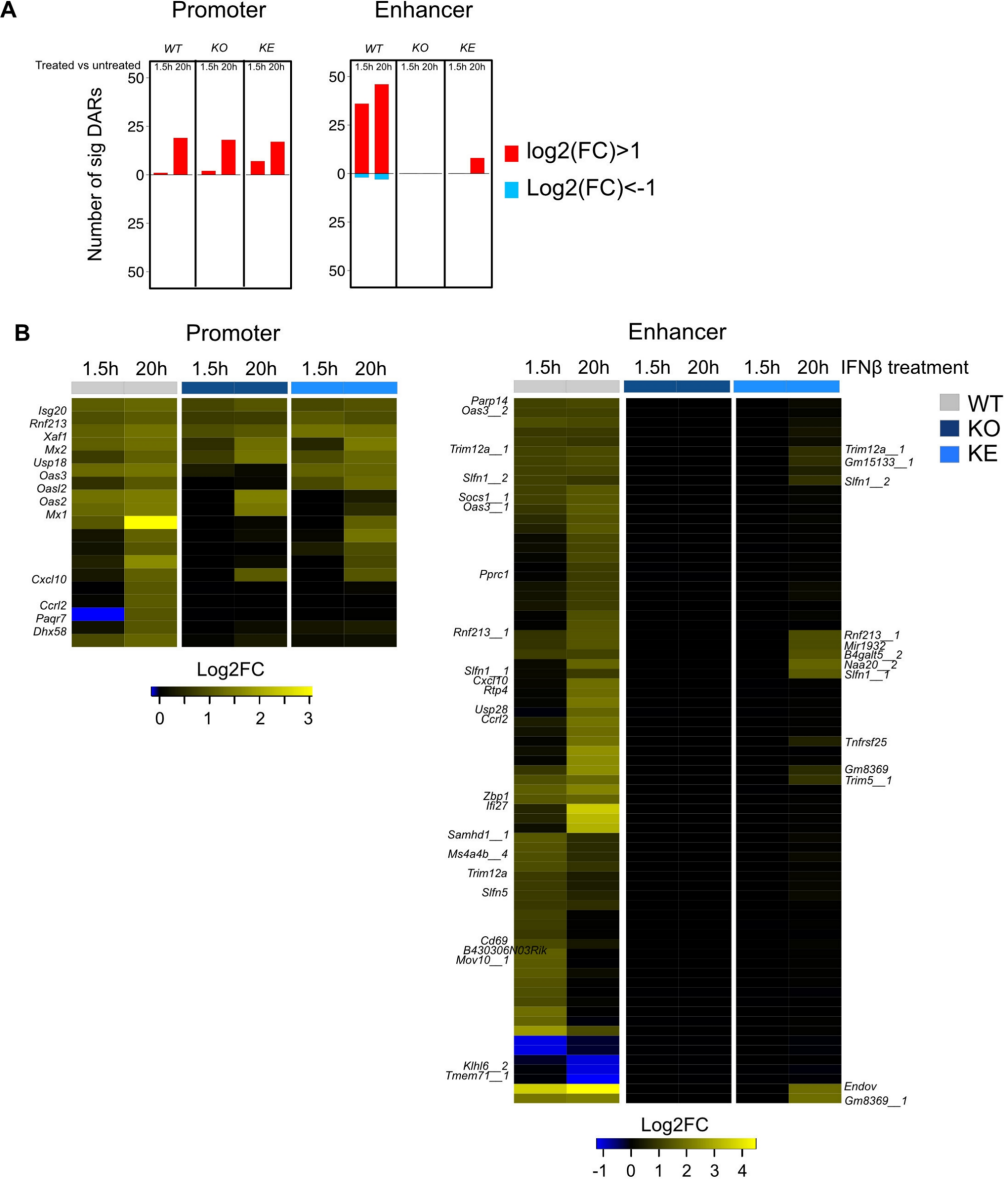
Chromatin accessibility of IFNβ-treated CD8^+^ T cells.** A** Number of significantly (|FC|> 2 or absolute log2(FC) > 1, FDR < 0.05) differentially accessible (DA) regions within promoter regions (± 200 bp from TSS) or proximal enhancer regions (± 3000 > bp < 200 bp from TSS) determined by DESeq2 in the indicated genotype comparisons; more accessible (red) and less accessible (blue). **B** Heatmap of log2(FC) of significantly DA regions in *WT* CD8^+^ T cells within promoter or enhancer regions

Recently we reported that IFNs rapidly increase chromatin accessibility at many response loci [98, 99, 112]. Among the immune cells analyzed, CD8^+^ T cells show the biggest kinase-inactive effect of TYK2 on the steady state and IFN-induced transcriptomes. We thus explored by ATAC-seq (assay for transposase-accessible chromatin with high-throughput sequencing) (i) whether the effect of the amplifier kinase TYK2 suffices to modify the chromatin architecture upon IFN treatment and (ii) whether the TYK2^K923E^ impact on DEG in CD8^+^ T cells translates into changes in chromatin structure. ATAC-seq data of in vitro cultured CD8^+^ T cells short-term and long-term treated with IFNβ or left untreated [27] were used to annotate the DA promoter regions (200 bp upstream and downstream of a transcription start site,TSS) and proximal promoter enhancers (3 kb upstream and downstream of TSS exclusive ± 200 bp). Using stringent significance criteria (FDR ≤ 0.05 and abs(log2FC) ≥ 1) the number of DA regions in all genotypes and treatment conditions was rather low. In *WT* cells at the promoter region the overall number of DA regions was lower than at the enhancer region and most changes became visible after long-term IFN treatment while at the enhancer region significant DA also appeared upon short-term treatment (Fig. 8A). At the promoter region neither TYK2 nor its kinase-inactive version are required for an increase of chromatin accessibility upon IFN stimulus, albeit the accessibility change is lower than in *WT* cells. This contrasts the enhancer regions where loss of TYK2 results in loss of chromatin changes and kinase-inactive TYK2 allows for some changes upon long-term IFN treatment (Fig. 8B right panel, loci indicated on the right side). Reduction of chromatin accessibility occurs only in *WT* cells, which is in line with the strict dependence of IRepG on *Tyk2*. The genes in *WT* cells showing congruent behavior in ATAC-seq and RNA-seq are indicated on the left side of the heatmaps (Fig. 8B and see above).

In summary, chromatin remodeling of IFN-treated CD8^+^ T cells in the absence of TYK2 grossly overlaps with the RNA signature: the effect of kinase-active TYK2 on chromatin accessibility at promoter regions upon IFN treatment is in line with the reduced but not abrogated transcriptional up-regulation of ISG and the absolute requirement of TYK2 for an IRepG signature. While at promoter-distant regions chromatin changes were abrogated in the absence of TYK2, at long-term IFN treatment TYK2 showed scaffolding functions at selected loci.

## Discussion

The JAK family member TYK2 is well known for its regulation of inflammation and immunity to infection and cancer. We recently reported that JAK-STAT signaling maintains homeostasis of immune cells and seems to be also crucial for the plasticity and vigilance of structural cells [27, 113, 114]. Here we investigate in detail the impact of TYK2 on common or cell-type-specific gene expression of macrophages, NK and CD8^+^ T cells under steady state, IFN-treated and tumor-derived conditions. To the best of our knowledge, we also provide the first detailed RNA signatures of kinase-inactive TYK2 in innate and adaptive splenic and tumor-derived immune cells.

Despite its engagement at various cytokine receptor complexes [6], the predominant impact of TYK2 in the investigated immune cells under homeostatic conditions and upon tumor infiltration is on IFN responses (Fig. 1, 7). This is in line with our previous observations of TYK2 effects in inflammatory macrophages [69]. Loss of TYK2 in mice and men leads to a partially reduced cytokine signaling strength at the TYK2-engaging receptors [4, 6] and to a substantial transcriptional decrease of STAT1 and other IFN response TFs (Fig. 3). The positive regulatory loop of the IFN signaling worsens the responsiveness to IFNs in TYK2-mutant cells compared to other cytokine responses with absent or less pronounced feed-forward loops.

As described previously [47, 114, 115], the baseline and induced IFN signatures are split in highly cell-type-specific and common ISG (Figs. 2 and 6). The common ISG show a robust and high amplitude of expression, are common to all mammals and include key regulators of the IFN feed-forward loop such as *Stat1*, *Irf7*, *Irf9* and *Usp18* and members of the *Oas*, *Ifit*, or *Mx* families [50, 51]. Note, that also robust detectable ISG show some degree of cell type specificity and TYK2-deficiency leads to variable degrees of responsiveness among ISG under steady state and challenged conditions (Figs. 2, 6, 7).

Mechanistically, the cell type specificity of the ISG signatures is governed by (i) the chromatin architecture, the cell fate commitment and the transcriptional memory leading to cell type-specific availability and/or usage of TFs [116–120], (ii) the differing exposure of cell types to para- and autocrine cytokines during their differentiation and in their respective splenic environment [121, 122], (iii) the cell type-specific feed-forward and feedback loops regulating key components of the JAK-STAT pathway [18, 116, 123], including unusual and/or non-phosphorylated STAT complexes [124–126], and (iv) the cell type variation in signaling pathway crosstalks and TF cooperativity [116, 127]. This leads to varying availabilities of positive and negative regulatory cytokine response modules in different cell types (Fig. 3). Of note, for splenic immune cells it is established that the chromatin architecture is shaped by basal, e.g. microbiome-induced, type I IFN that acts at a distance and maintains their alertness [128, 129]. Depending on their spatial localization, splenic cells may be exposed to different type I IFN levels [70].

Our recent data emphasize that the impact of JAK-STAT on homeostatic and induced transcriptomes results not only from positive control of gene expression, but also from gene repression [27]. In contrast to the transcriptional amplification, the repressive functions upon IFN stimulus are completely TYK2-dependent in NK and CD8^+^ T cells (Fig. 6). The underlying mechanisms are the known negative feedback loops of IFN signaling [130] and also direct effects at promoter level such as the displacement of activating factors or the recruitment of chromatin modifiers [131, 132]. Our promoter analysis of IRepG revealed an overrepresentation of zink finger binding proteins but no enrichment for classical ISRE/GAS elements (Fig. 6C). The latter is confirmed by the pioneering study on IRepG [49] and suggests other DNA-binding proteins or alternate STAT1-containing complexes suppress gene expression during early and late IFN responses. Mechanistically, the strict TYK2/STAT1 dependency indicates that the transcriptional repressor(s) are IFN-inducible genes and capable of binding GC-rich elements and SP TF family motifs in the proximity of IRepG. Candidates for these criteria are TF of the BCL, IRF, KLF, NFY and PRDM families, which are among the IFN-dependent DEG (Table S2). In line with this, a previous proteomics study in human cells suggested SP1, IRF2, PRDM1 and BCL6 as candidate regulators of IRepG [102].

We have shown previously that the TYK2 function in NK cell maturation is cell extrinsic and independent of IFNAR signaling in these cells [82, 133]. With respect to quantity and quality of gene expression, NK cells show a high degree of TYK2 requirement (Figs. 1, 4 and 7). The DEG signatures in kinase-inactive NK cells give little indication of a restoration of maturation or cytotoxicity at the level of transcription, neither in naïve nor in tumor-derived cells (Figs. 4 and 7). It seems unlikely that other JAK family members compensate for the lack of TYK2 enzymatic activity. We found no evidence for an alteration of STAT activation in our previous studies: Primary cells from *Tyk2*^*K923E*^ and *Tyk2*^−/−^ mice show similarly impaired cytokine-induced STAT phosphorylation [8, 30]. Lack of restoration or alternative STAT activation by kinase-inactive TYK2 was also reported in studies by others employing genetically modified cells and mice [3, 5, 9], human patients ([2, 3]) and pharmacologic kinase inhibitors [134]. Basal mitochondrial activity, which was reported to be independent of TYK2 kinase activity in B cells [89], appears similarly impaired in *Tyk2*^−/−^ and *Tyk2*^*K923E*^ NK cells (Fig. 4). We therefore suggest that the observed anti-tumor activity of kinase-inactive NK cells originates from crosstalks with other cells showing altered transcriptional programs or more likely from NK cell-intrinsic and/or -extrinsic post-transcriptional activities. Along that line it is notable, that we have reported post-transcriptional activities of TYK2 [135] and translational control mechanisms of ISG [136, 137] and immune cells [138, 139] are well established.

CD8^+^ T cells of *Tyk2*^*K923E*^ mice show no restoration of cytolytic activity (Fig. 5) and neither naïve nor tumor-challenged transcriptomes show signatures switches indicating clear functional implications (Figs. 5 and 7). Nevertheless, the number of *Tyk2*^*K923E*^-dependent DEG in these cells is the highest (Fig. 1B) and the scaffolding functions of TYK2 lead to chromatin accessibility at promoter-distal regions upon cytokine treatment (Fig. 8).

Considering the high physiological similarities between mice and men concerning TYK2 deficiency [4, 24, 140], loss of TYK2 enzymatic activity [3, 30] and genetic versus pharmacologic TYK2 inhibition [67, 134] our data are also relevant in light of the patient cohort with inborn TYK2 impairments [141] and the treatment of psoriasis and several ongoing clinical trials for other autoimmune and inflammatory diseases with respect to hitherto overseen tissue and cell-type-specific effects and potential epigenomic and post-transcriptional effects [142–144].

Our study unravels the transcriptional impact of TYK2 in naïve immune cells and upon exposure to a tumor microenvironment. In addition, we identify an unambiguous role of TYK2 in driving the suppression of distinct gene sets upon IFN treatment. We propose to further elucidate the post-transcriptional effects of kinase-inactive TYK2.

## Supplementary Information

Below is the link to the electronic supplementary material.

Supplementary file1 (XLSX 12 KB)

Supplementary file2 (XLSX 961 KB)

Supplementary file3 (XLSX 287 KB)

Supplementary file4 (XLSX 26 KB)

Supplementary file5 (XLSX 157 KB)

Supplementary file6 (XLSX 2513 KB)

Supplementary file7 (DOCX 4022 KB)

## Data Availability

Raw and processed RNA-seq and ATAC-seq data are available from the NCBI Gene Expression Omnibus (GEO) repository (Accession Numbers: GSE204736, GSE274337, GSE281132, GSE281133). The Supplementary Website (http://jakstat.bocklab.org) provides data links and genome browser tracks for interactive data visualization. Genome assemblies and gene annotations (mm10/GRCm38 release 93) are available from Ensembl (https://ensembl.org). The complete list of genes analyzed, their fold change and significance and compiled lists of reference genes can be obtained in the Supplementary Tables.

## Abbreviations

ATAC: Assay for transposase-accessible chromatin
CTL: Cytotoxic T lymphocyte
DA: Differential accessibility
DE: Differential expression
DEG: Differentially expressed gene
GSEA: Gene set enrichment analysis
IFN: Interferon
IL: Interleukin
IRepG: IFN-repressed gene
IRF: IFN regulatory factor
ISG: IFN-stimulated gene
ISRE: IFN-stimulated response element
JAK: Janus kinase
NES: Normalized enrichment score
SOCS: Suppressor of cytokine signaling
STAT: Signal transducer and activator of transcription
TF: Transcription factor
TYK2: Tyrosine kinase 2
WT: Wild type
