# Supplementary material for: Cell-type-specific requirement for TYK2 in murine immune cells under steady state and challenged conditions
Source: Cell Mol Life Sci. 2025 Mar 2;82(1):98. doi: 10.1007/s00018-025-05625-9 (PMC11872851; doi:10.1007/s00018-025-05625-9)
Supplement: Supplementary file 7 — Supplementary file7 (DOCX 4022 KB) [file 18_2025_5625_MOESM7_ESM.docx]

**Supplemetary Figures for**

**Cell-type-specific requirement for TYK2 in murine immune cells under steady state and challenged conditions**

Anzhelika Karjalainen^1^, Agnieszka Witalisz-Siepracka^1,2^, Michaela Prchal-Murphy^3^, David Martin^1^, Felix Sternberg^4,5^, Milica Krunic^6^, Marlies Dolezal^7^, Nikolaus Fortelny^8^, Matthias Farlik^9^, Sabine Macho-Maschler^1^, Caroline Lassnig^10^, Katrin Meissl^1^, Lena Amenitsch^1^, Therese Lederer^1^, Elena Pohl^4^, Dagmar Gotthardt^2^, Christoph Bock^11^, Thomas Decker^12^, Birgit Strobl^1^, Mathias Müller^1^*

*** Correspondence:** Corresponding Author [mathias.mueller@vetmeduni.ac.at](mailto:mathias.mueller@vetmeduni.ac.at) (https://orcid.org/0000-0002-7879-3552)


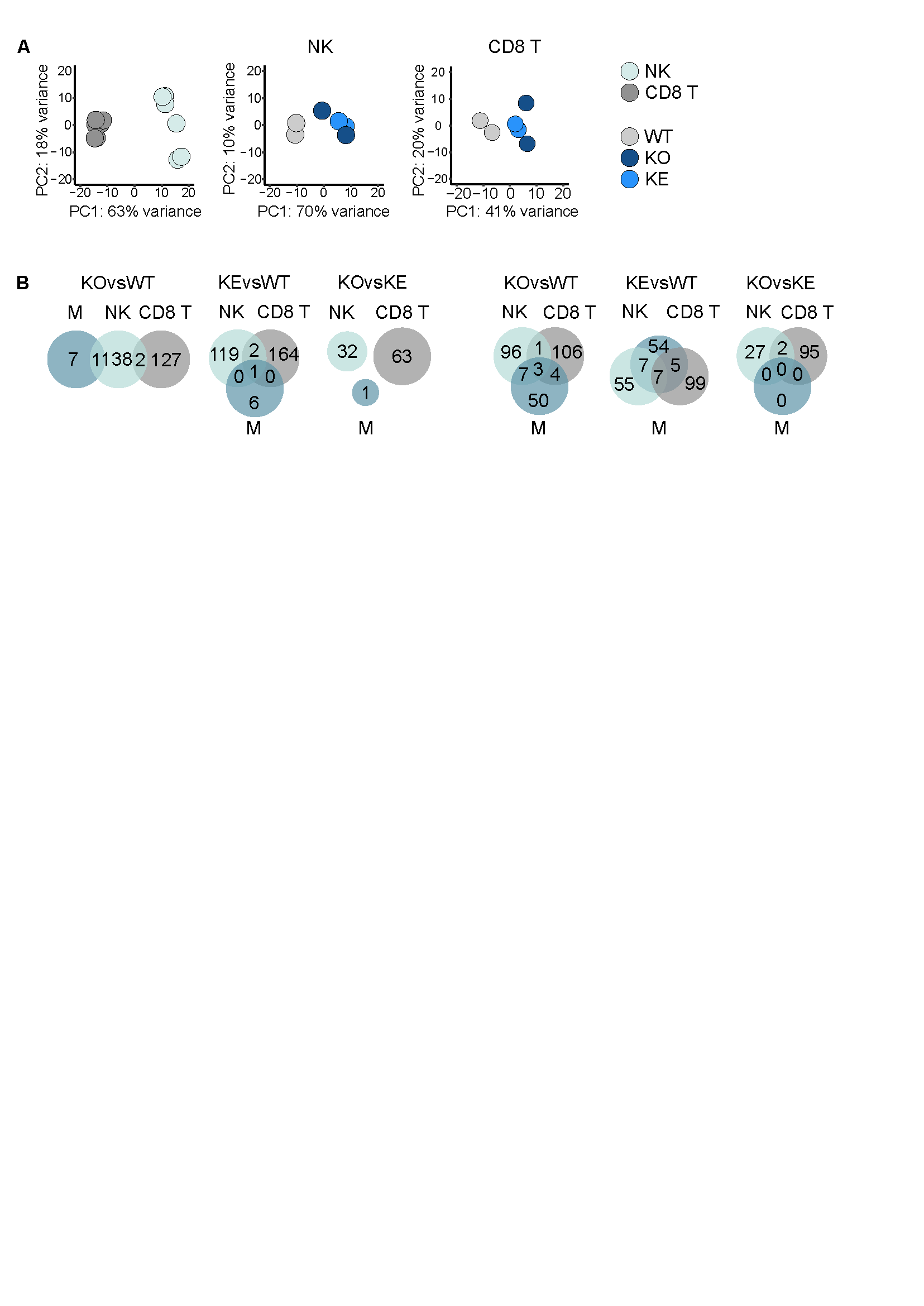


**Fig. S1. Principal component analyses and determination of common up- and down-regulated DEG**

**A** Two-dimensional representation of the similarity of transcriptional effects of cell types and genotypes based on principal component analysis (PCA). **B** Venn diagrams of genotype comparisons and cell type-specific up- and downregulated DEG.


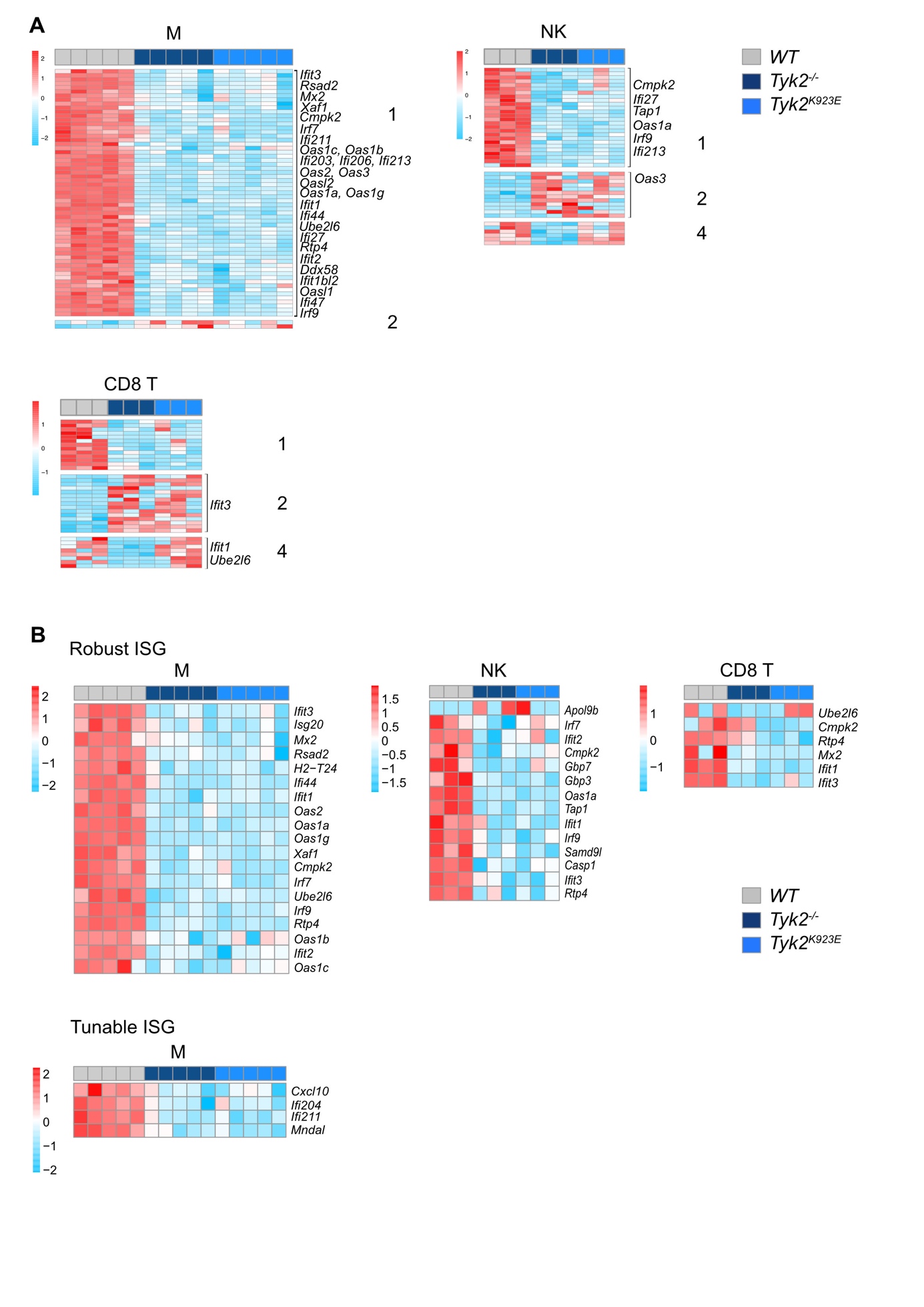


**Fig. S2. ISG signatures under steady state conditions in *Tyk2*-mutated cell types**

**A** Heat maps of splenic macrophages (M), NK cells and CD8^+^ T cells with genotype specificity of DE ISG, respective genotype patterns of DEG (1, 2, 4) in Fig. 1C are indicated. **B** Scaled mRNA expression values of selected ISG known to be robust expressed or to undergo minor transcriptional changes (tunable).


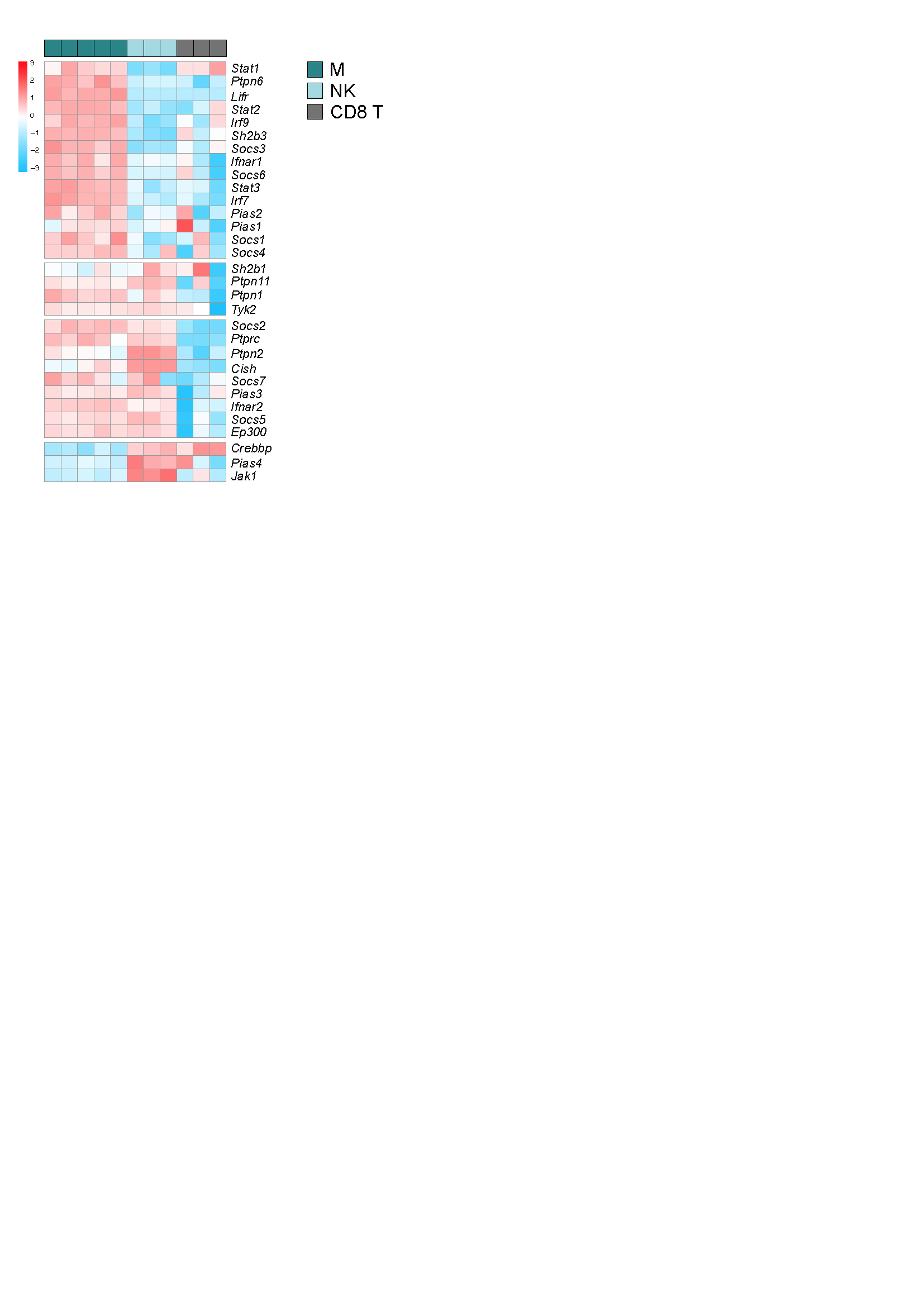


**Fig. S3. JAK-STAT signaling components in *WT* cells under steady state condition**

Scaled mRNA expression values of JAK-STAT signaling components in *WT* macrophages (M), NK and CD8+ T cells.


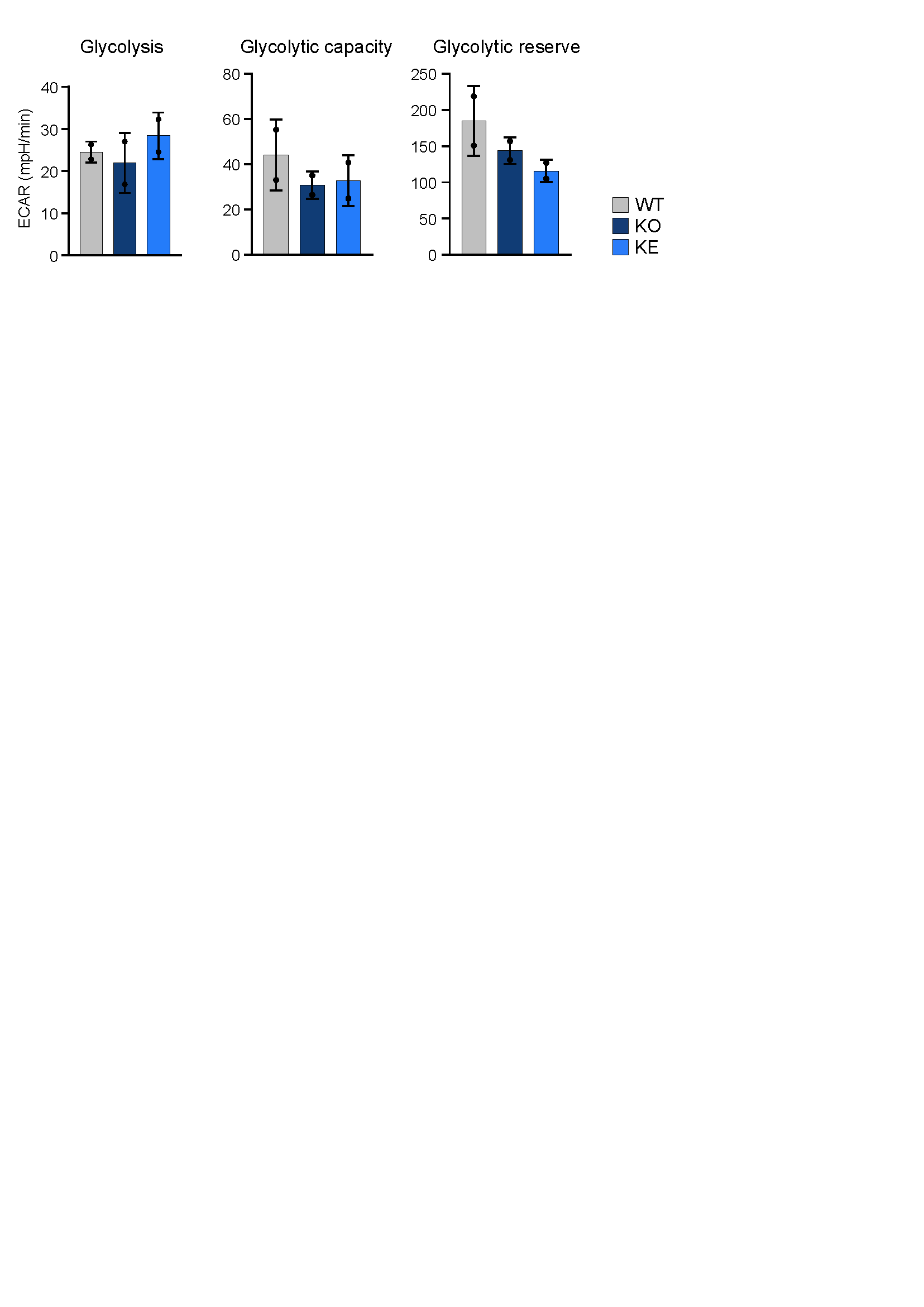


**Fig. S4. Glycolytic performance of *Tyk2*-mutant NK cells**

Agilent Seahorse XF Glycolysis Stress Test of NK cells. NK cell glycolysis, glycolytic capacity and glycolytic reserve were calculated for *WT*, *Tyk2^-/-^* and *Tyk2^K923E^* cells (n = 2). Two-way ANOVA with Tukey multiple comparison was used to assess statistical significance, *p ≤ 0.05. ECAR: extracellular acidification rate.


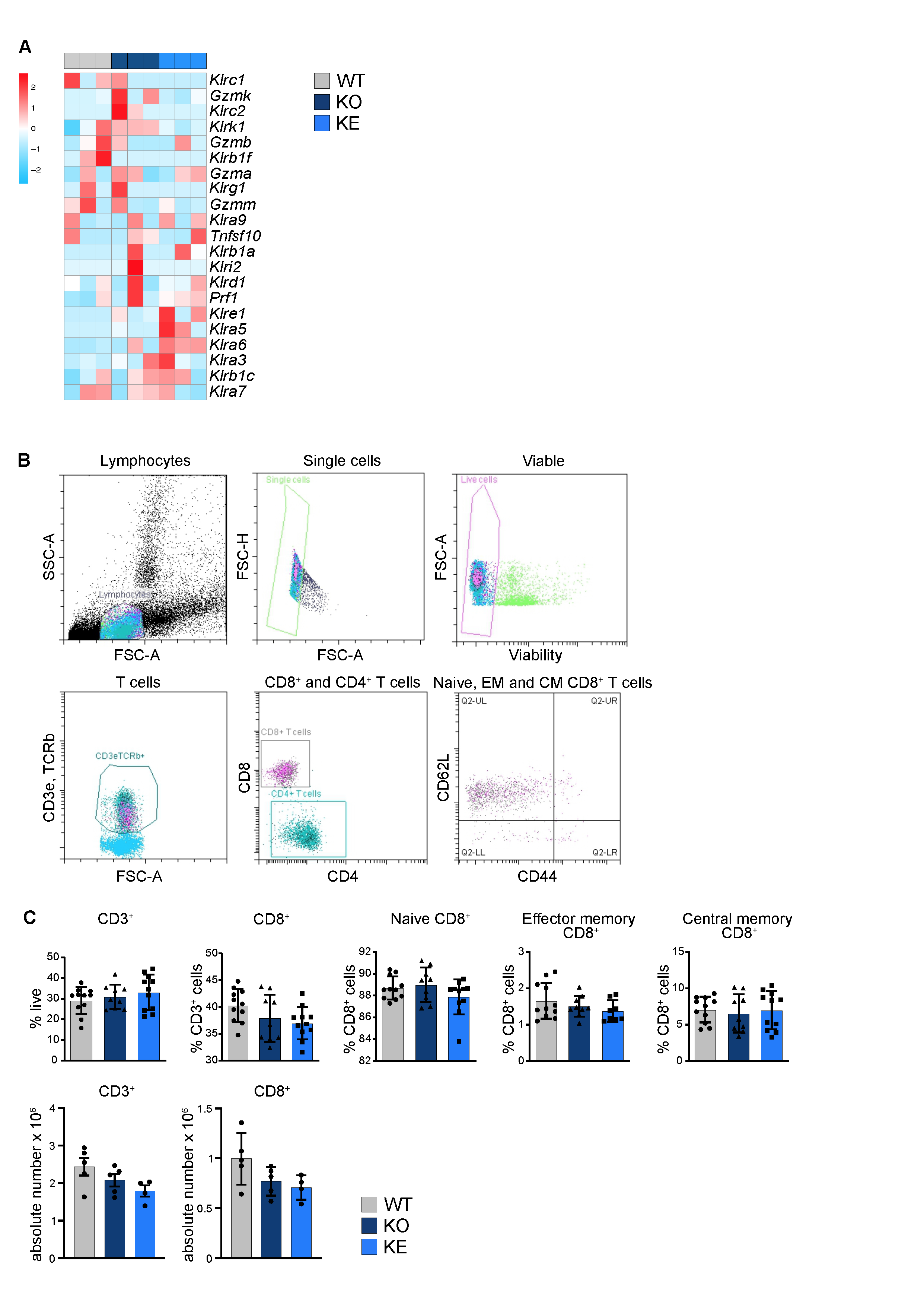


**Fig. S5. Effector gene expression and characterization of CD8^+^ T cell subsets**

**A** Scaled mRNA expression values of selected effector genes. **B** Splenocytes from WT, Tyk2^-/-^, and Tyk2^K923E^ mice were isolated, and CD8^+^ T cell subsets were identified by flow cytometry. **C** The distribution of naïve (CD62L^+^ CD44^-^), central memory (CM, CD62L^+^ CD44^+^) and effector memory (EM, CD62L^-^ CD44^+^) CD8^+^ T cells in splenic T cells. The absolute number of CD3^+^ T cells and CD8^+^ CD3^+^ T cells were calculated from the percentage of the living cells. Mean values ± SD of biological replicates from two independent experiments are shown: n=9-11 per group. * p < 0.05.


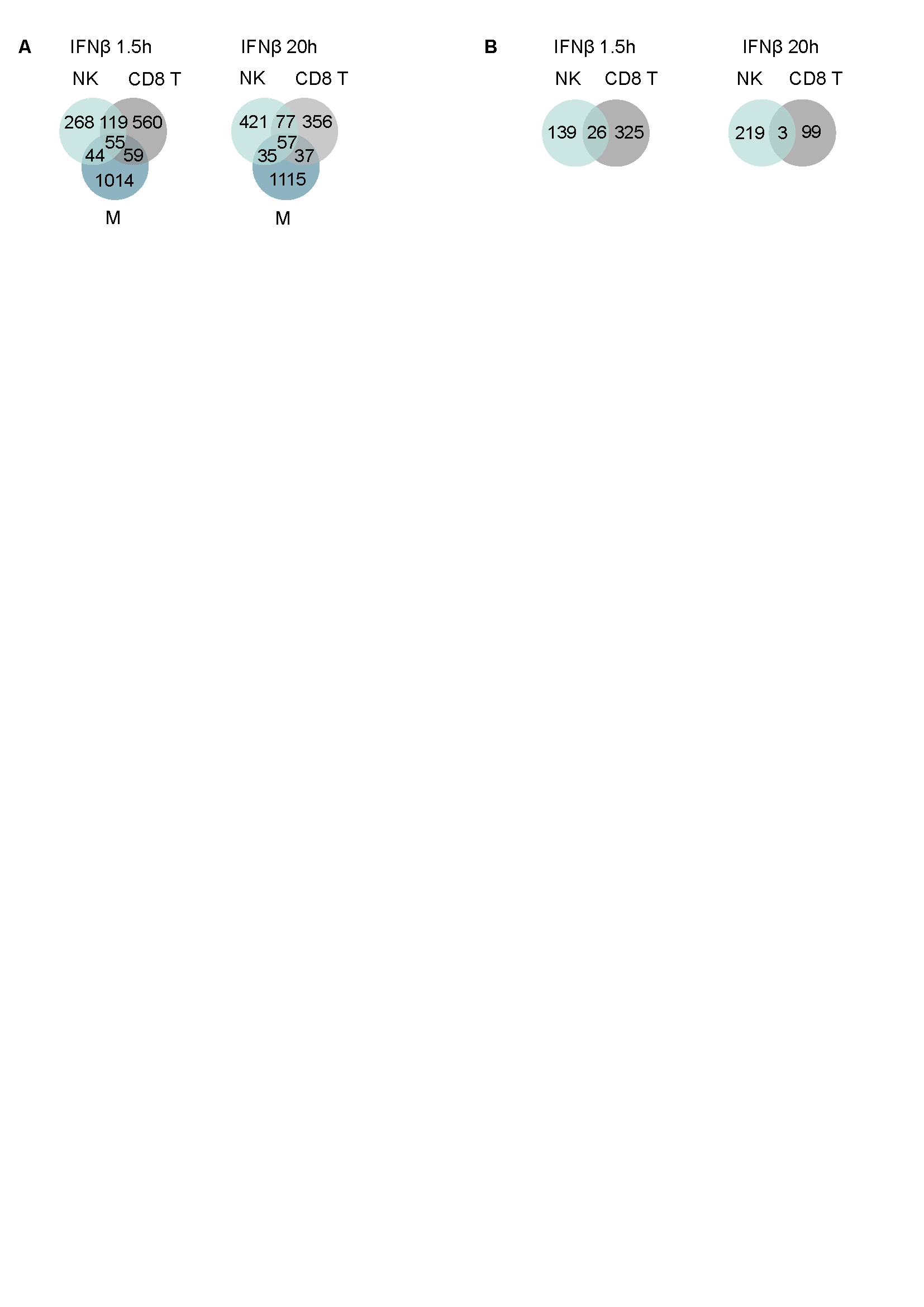


**Fig. S6. Cell type specificity of IFNβ challenges**

Venn diagrams of cell type-specific early (1.5h treatment) or late (20h treatment) **A** IFNβ-upregulated and **B** downregulated genes.


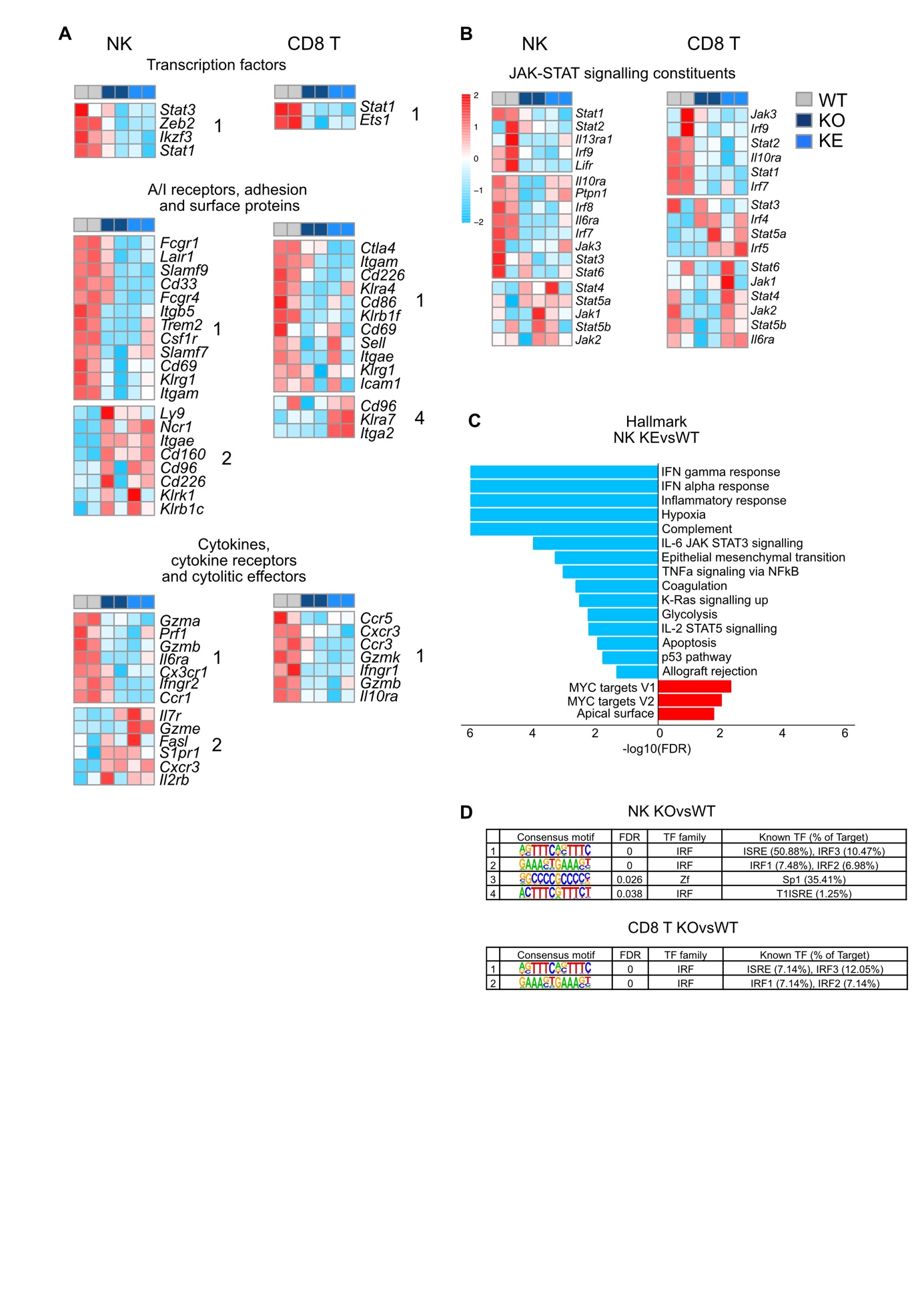


**Fig. S7. Transcriptional profiles of effector genes and JAK-STAT signaling components in cytolytic tumor-infiltrating cells**

**A** Scaled mRNA expression values of selected effector genes (log2 cpm, counts per million); A/I, activating/inhibitory; genotype patterns of DEG (1-2) are indicated. **B** Scaled mRNA expression values of JAK-STAT signaling constituents (log2 cpm, counts per million). **C** Gene set enrichment analysis (GSEA) of NK or CD8^+^ T cell DEG from indicated comparisons; positive normalized enrichment scores (NES) (red) and negative NES (blue). **D** Hypergeometric Optimization of Motif EnRichment (HOMER) cis-regulatory motif discovery of DEG from indicated comparisons of tumor-infiltrating NK and CD8^+^ T cells; FDR, transcription factor (TF) family and members as well as the percent of genes with the enriched motifs are indicated.
